# Limited nutrient availability in the tumor microenvironment renders pancreatic tumors sensitive to allosteric IDH1 inhibitors

**DOI:** 10.1038/s43018-022-00393-y

**Published:** 2022-06-09

**Authors:** Ali Vaziri-Gohar, Joel Cassel, Farheen S. Mohammed, Mehrdad Zarei, Jonathan J. Hue, Omid Hajihassani, Hallie J. Graor, Yellamelli V. V. Srikanth, Saadia A. Karim, Ata Abbas, Erin Prendergast, Vanessa Chen, Erryk S. Katayama, Katerina Dukleska, Imran Khokhar, Anthony Andren, Li Zhang, Chunying Wu, Bernadette Erokwu, Chris A. Flask, Mahsa Zarei, Rui Wang, Luke D. Rothermel, Andrea M. P. Romani, Jessica Bowers, Robert Getts, Curtis Tatsuoka, Jennifer P. Morton, Ilya Bederman, Henri Brunengraber, Costas A. Lyssiotis, Joseph M. Salvino, Jonathan R. Brody, Jordan M. Winter

**Affiliations:** 1 Case Comprehensive Cancer Center, Case Western Reserve University, Cleveland, OH USA; 2 Molecular and Cellular Oncogenesis Program, The Wistar Institute, Philadelphia, PA USA; 3 Department of Surgery, Division of Surgical Oncology, University Hospitals, Cleveland Medical Center, Cleveland, OH USA; 4 Cancer Research UK Beatson Institute, Glasgow, UK; 5 Department of Genetics and Genome Sciences, Case Western Reserve University, Cleveland, OH USA; 6 Department of Nutrition, Case Western Reserve University, Cleveland, OH USA; 7 Jefferson Pancreas, Biliary and Related Cancer Center, Thomas Jefferson University, Philadelphia, PA USA; 8 Department of Molecular and Integrative Physiology, University of Michigan School of Medicine, Ann Arbor, MI USA; 9 Department of Radiology, Case Western Reserve University, Cleveland, OH USA; 10 Deptartments of Radiology, Biomedical Engineering, and Pediatrics, Case Western Reserve University, Cleveland, OH USA; 11 Department of Veterinary Physiology and Pharmacology, Texas A&M University, College Station, TX USA; 12 Department of Physiology and Biophysics, Case Western Reserve University, Cleveland, OH USA; 13 Code Biotherapeutics Inc, Hatfield, PA USA; 14 Department of Population and Quantitative Health Sciences, Case Western Reserve University, Cleveland, OH USA; 15 Institute of Cancer Sciences, University of Glasgow, Glasgow, UK; 16 Department of Nutrition and Biochemistry, Case Western Reserve University, Cleveland, OH USA; 17 Brenden Colson Center for Pancreatic Care; Departments of Surgery and Cell, Developmental & Cancer Biology; Knight Cancer Institute, Oregon Health and Science University, Portland, OR USA

**Keywords:** Cancer therapy, Cancer microenvironment, Cancer metabolism, Cancer

## Abstract

Nutrient-deprived conditions in the tumor microenvironment (TME) restrain cancer cell viability due to increased free radicals and reduced energy production. In pancreatic cancer cells a cytosolic metabolic enzyme, wild-type isocitrate dehydrogenase 1 (wtIDH1), enables adaptation to these conditions. Under nutrient starvation, wtIDH1 oxidizes isocitrate to generate α-ketoglutarate (αKG) for anaplerosis and NADPH to support antioxidant defense. In this study, we show that allosteric inhibitors of mutant IDH1 (mIDH1) are potent wtIDH1 inhibitors under conditions present in the TME. We demonstrate that low magnesium levels facilitate allosteric inhibition of wtIDH1, which is lethal to cancer cells when nutrients are limited. Furthermore, the Food & Drug Administration (FDA)-approved mIDH1 inhibitor ivosidenib (AG-120) dramatically inhibited tumor growth in preclinical models of pancreatic cancer, highlighting this approach as a potential therapeutic strategy against wild-type IDH1 cancers.

## Abstract (web summary)

Winter and colleagues demonstrate that metabolic adaptation to nutrient stress in the tumor microenvironment of pancreatic cancer leads to a dependency on IDH1, which is therapeutically actionable regardless of *IDH1* mutation status.

## Main

Pancreatic cancer (pancreatic ductal adenocarcinoma, PDAC) cells adapt to austere conditions created by a dense and hypovascular stroma in the TME^1–6^. These same adaptive survival pathways protect pancreatic cancer cells against chemotherapy^7^. Thus, the best available treatments against PDAC (that is, chemotherapy) are less effective under tumor-associated conditions. Investigative pursuits that identify metabolic dependencies in primary and metastatic pancreatic cancer^1,8–15^ should reveal attractive therapeutic alternatives that attack biologic vulnerabilities, especially in the context of the TME. Examples of relevant biologic processes utilized by PDAC cells to overcome nutrient limitation include autophagy^16^, macropinocytosis^3,17^ and the utilization of secreted alanine from pancreatic stellate cells^18^. A growing body of evidence shows that mitochondrial function and antioxidant defense are also crucial under low nutrient conditions. When energy substrates are scarce, oxidative phosphorylation is needed to maximize ATP generation^19,20^ and nutrient limitation is highly oxidative because glucose is the principal substrate for NADPH synthesis^21–23^.

We previously determined that an RNA-binding protein, human antigen R (HuR; ELAVL1), enhanced both antioxidant defense and mitochondrial function under nutrient withdrawal. HuR accomplishes this in part through post-transcriptional stabilization of wtIDH1. Out of 40 antioxidant enzymes, only IDH1 expression was lost in multiple HuR-knockout cell lines, pointing to an HuR–*IDH1* regulatory axis as a key component of the acute antioxidant response to stress^2,24^. Additional studies identified the regulatory HuR binding site on the IDH1 3'-UTR^7^.

IDH1 is a cytosolic enzyme that catalyzes the reversible conversion of isocitrate and αKG. The reaction uses NADP^+^ or NADPH as a cofactor, depending on the direction of the reaction^25–27^. Surprisingly few studies have focused on the role of wtIDH1 in cancer cell survival and tumor growth^7,25–31^, and these studies generally did not consider the reliance of cancer cells on wtIDH1 in the context of nutrient limitation, which is a key feature of tumors^7^. However, this cancer-associated stress is relevant since studies suggest that glucose may be more limiting than oxygen within the TME^32^. Based on our previous studies revealing *IDH1* as a regulatory target of HuR, and the enzyme’s direct role in the generation of NADPH and αKG through the oxidation of isocitrate, we hypothesized that wtIDH1 is essential for PDAC cells under metabolic stress. More specifically, we suspected that the products of the oxidative wtIDH1 reaction, NADPH and αKG, directly power antioxidant defense and mitochondrial function to promote PDAC survival. Herein, we also demonstrate that compounds developed to target mutant IDH1 can be repurposed as wild-type IDH1 inhibitors, because these drugs surprisingly become potent inhibitors of wtIDH1 in cancer cells under conditions present in tumors: specifically, we found that wtIDH1 is critical for PDAC cell survival under low-glucose conditions, and allosteric IDH inhibitors effectively block wtIDH1 activity under low magnesium.

## Results

### IDH1 protects PDAC cells from oxidative stress under nutrient limitation

**Fig. 1:**
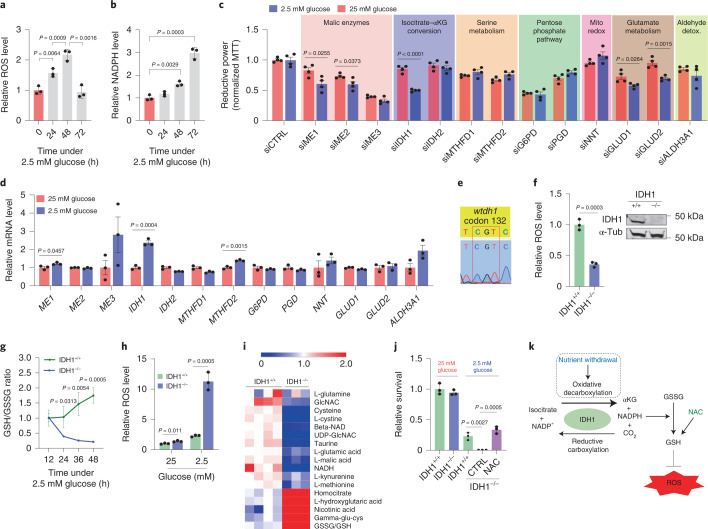
IDH1 supports antioxidant defense under nutrient withdrawal in pancreatic cancer (MiaPaCa-2) cells. **a**, ROS levels were detected by DCFDA assay under glucose withdrawal (2.5 mM) over 72 h. 0 h indicates that cells were incubated under standard, 25 mM glucose (*n* = 3 independent biological replicates). **b**, NADPH levels under the indicated conditions over 72 h (*n* = 3 independent biological replicates). **c**, Reductive power, as detected by MTT assay normalized to cell number. Cells were transiently transfected with siRNAs against different NADPH-generating enzymes and incubated under the indicated conditions for 72 h (*n* = 4 independent biological replicates). **d**, Relative transcripts per million values for NADPH-generating transcripts under the indicated conditions for 48 h (*n* = 3 individual biological replicates). **e**, Sanger sequencing of amplicons correlating with codon 132 of the *wtIDH1* gene. The reference human wild-type sequence is shown. **f**, Immunoblot of IDH1 in IDH1^+/+^ and IDH1^–/–^ MiaPaCa-2 PDAC cells (representative immunoblots of three biological replicates with similar results are shown) and relative NADPH levels (*n* = 3 independent experiments). **g**,**h**, reduced glutathione/oxidized glutathione (GSH/GSSH) ratio (**g**) and ROS levels (**h**) under the indicated conditions for 48 h (*n* = 3 independent biological replicates). **i**, Redox-related metabolites in IDH1^+/+^ and IDH1^–/–^ cells under glucose withdrawal (2.5 mM) for 48 h (IDH1^+/+^, *n* = 4; IDH1^–/–^, *n* = 3 individual biological replicates). **j**, Relative clonogenic growth of indicated cells treated with or without glutathione precursor NAC under the indicated conditions. NAC treatment (1.25 mM) was given 16 h before cell culture under low-glucose conditions (*n* = 3 independent biological replicates). **k**, Enzymatic reaction of IDH1. Data provided as mean ± s.d. (**a**,**b**,**f**–**h**,**j**) or mean ± s.e.m. (**c**). Pairwise comparisons were conducted using two-tailed, unpaired Student’s *t*-tests. CTRL, control. Source data

**Extended Data Fig. 1:**
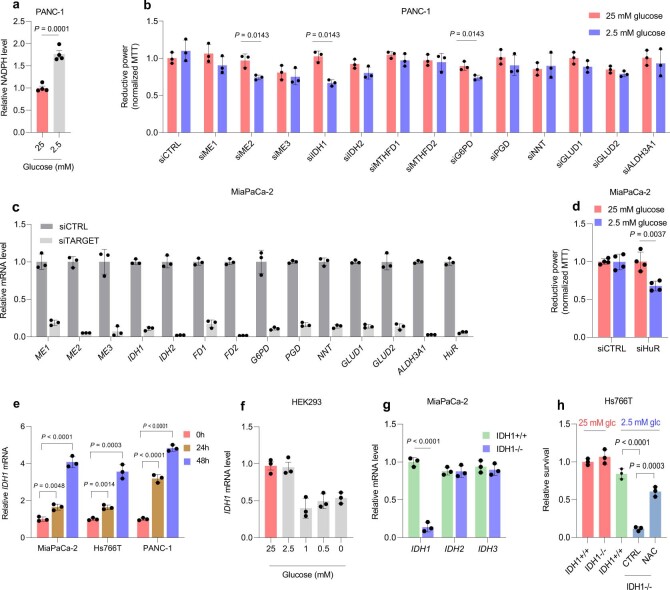
Validation of IDH1s’ role in redox balance in pancreatic cancer cells. **a**, NADPH levels in PANC-1 pancreatic cancer cells under the indicated conditions for 72 hours (n = 4 independent biological replicates). **b**, Reductive power, as measured by an MTT assay normalized to cell number, after transient transfection of siRNAs against NADPH-generating enzymes in PANC-1 cells. Cells were incubated under the indicated conditions for 72 hours (n = 3 independent biological replicates). **c**, mRNA levels associated with siRNA screening shown in Fig. 1c (a representative of three independent biological replicates with similar results is shown). **d**, HuR in MiaPaCa-2 cells as a positive control (n = 4 independent biological replicates). **e**, qPCR analysis of *IDH1* mRNA normalized to 18 S in multiple PDAC cells and under 2.5 mM glucose for the indicated time intervals (n = 3 independent biological replicates). **f**, qPCR analysis of *IDH1* mRNA normalized to 18 S in HEK293 human embryonic kidney cells cultured under different concentrations of glucose for 48 hours (n = 3 independent biological replicates). **g**, mRNA quantitation in MiaPaCa-2 cells by qPCR of *IDH1*, *IDH2* and *IDH3* transcripts normalized to 18 S (n = 3 independent biological replicates). **h**, Relative clonogenic growth of Hs766T cells with or without NAC (1.25 mM) for five days at the indicated conditions (n = 3 independent biological replicates). Data are provided as mean ± s.d. (**b**,**c**,**e**-**h**) or mean ± s.e.m. (**a**,**d**) or. Pairwise comparisons were conducted using two-tailed, unpaired Student’s *t*-tests. Source data

Pancreatic ductal adenocarcinoma cells were cultured under low-glucose conditions (2.5 mM) to simulate the levels present in the pancreatic cancer microenvironment^3^. A surge in reactive oxygen species (ROS) levels (Fig. 1a) occurred over 48 h, then PDAC cells compensated by a rise in NADPH (Fig. 1b and Extended Data Fig. 1a). Consequently, ROS levels returned to baseline within 3 days (Fig. 1a), illustrating effective adaptation and redox homeostasis under glucose withdrawal. To investigate the role of NADPH-generating enzymes in this adaptive response, a small interfering RNA screen of all 13 NADPH-generating enzymes was performed. Only IDH1 silencing reproducibly lowered reductive power under glucose withdrawal (2.5 mM glucose for 72 h) in two different PDAC cell lines (MTT assay, normalized to cell number; Fig. 1c and Extended Data Fig. 1b,c). HuR silencing was used as a positive control for ROS induction under glucose withdrawal^7^ (Extended Data Fig. 1d). This unbiased siRNA screen complements the aforementioned and independent screen of antioxidant enzymes, revealing HuR’s regulatory control of IDH1 (refs. ^7,30^). Together, these experiments point to IDH1 as an enzyme critical for pancreatic cancer adaptation to oxidative stress. The role of IDH1 as an acute stress response and survival protein was further demonstrated by the rapid rise in *IDH1* messenger RNA expression upon glucose withdrawal, presumably related to stabilization by HuR^7^, and across multiple PDAC cell lines (Extended Data Fig. 1e). The enzyme proved to be the only NADPH-generating enzyme that responded acutely in this way (Fig. 1d). Notably, PDAC cells were genotyped and revealed wild-type IDH1 sequence (Fig. 1e). In contrast to findings with PDAC cells, IDH1 upregulation did not occur in a noncancer cell line cultured under glucose withdrawal (Extended Data Fig. 1f), raising the possibility that this enzyme is particularly important to cancer cells. Two different IDH1-knockout PDAC cell lines (MiaPaCa-2 and Hs766T IDH1^–/–^) were generated via CRISPR–Cas9 gene editing. *IDH2* and *IDH3* mRNA expression was similar between IDH1^−/−^ and isogenic controls (IDH1^+/+^) (Extended Data Fig. 1g), revealing a lack of compensation by the other IDH isoforms.

Initial studies of these cells tested the effects of IDH1 deletion on redox homeostasis. As observed with parental PDAC cells (Fig. 1a), control IDH1^+/+^ cells adapted well to oxidative stress from glucose withdrawal as reflected by stable NADPH (Fig. 1f) and glutathione (GSH) levels (Fig. 1g). However, IDH1^−/−^ cells failed to compensate effectively in these experiments. Similarly, ROS levels spiked in IDH1^−/−^ cells cultured under glucose withdrawal, as detected by increased dichlorodihydrofluorescein diacetate (DCFDA) (Fig. 1h). The results of an unbiased metabolomics screen were consistent with these findings (Fig. 1i). After 48 h of glucose withdrawal, oxidized metabolites such as oxidized glutathione (GSSG) were substantially elevated in IDH1^−/−^ cells. IDH1 knockout had no effect on cell viability in an in vitro clonogenic assay performed under glucose abundance (for example, 25 mM). However, IDH1^–/–^ cells derived from multiple PDAC cell lines failed to survive glucose withdrawal. Antioxidants such as *N*-acetyl-cysteine (NAC, a glutathione precursor) rescued IDH1^−/−^ cells under these oxidizing conditions (Fig. 1j and Extended Data Fig. 1h). Taken together, these data indicate that PDAC cells rely on IDH1-dependent oxidative decarboxylation for redox balance under metabolic stress (Fig. 1k).

### IDH1 supports mitochondrial function under metabolic stress

**Fig. 2:**
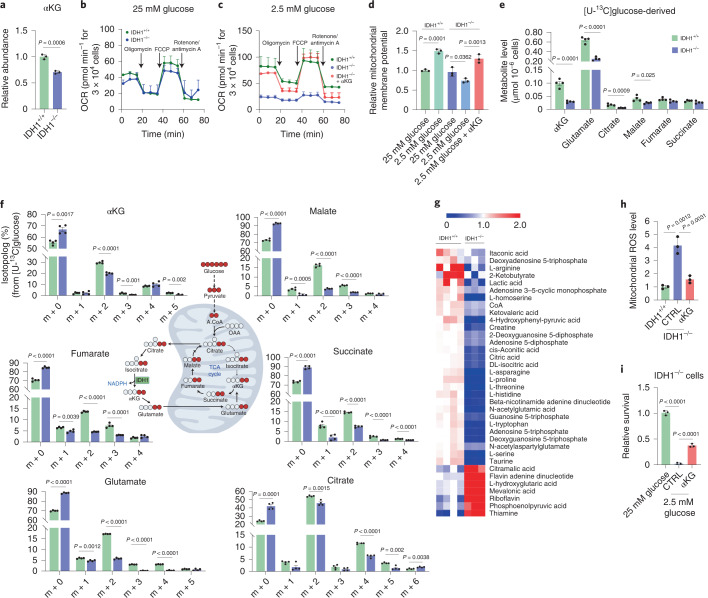
IDH1 supports mitochondrial function under nutrient withdrawal in pancreatic cancer (MiaPaCa-2) cells. **a**, Relative abundance of αKG in cells under low-glucose conditions (2.5 mM glucose) for 48 h (*n* = 3 independent biological replicates). **b**,**c**, OCR in MiaPaCa-2 pancreatic cancer cells under 25 mM (**b**) and 2.5 mM glucose (**c**). In a rescue experiment, cells were treated initially with αKG (4 mM) for 6 h and glucose was then lowered to 2.5 mM for 30 h (representative experiments of three independent biological replicates with similar results are shown). **d**, Mitochondrial membrane potential measured by TMRE assay in cells under the indicated conditions for 30 h (*n* = 3 independent biological replicates). **e**,**f**, Total pool size, including glucose-independent (m + 0) and glucose-dependent isotopologs (**e**) and isotopolog distribution (**f**) in cells cultured with unlabeled 2.5 mM glucose for 38 h followed by 2.5 mM [U-^13^C]glucose for an additional 10 h (*n* = 4 individual biological replicates). **g**, Mitochondrial-related metabolites in IDH1^+/+^ and IDH1^–/–^ cells under glucose withdrawal for 48 h (IDH1^+/+^, *n* = 4; IDH1^–/–^, *n* = 3 individual biological replicates). **h**,**i**, Mitochondrial ROS levels in cells under low-glucose condition for 48 h (**h**) and relative clonogenic growth of IDH1^–/–^ cells treated with or without αKG (4 mM) (**i**) under the indicated conditions for 5 days (*n* = 3 independent biological replicates). Data are provided as mean ± s.d. (**a**–**d**,**h,i**) or mean ± s.e.m. (**e**,**f**). Pairwise comparisons were conducted using two-tailed, unpaired Student’s *t*-tests. Source data

**Extended Data Fig. 2:**
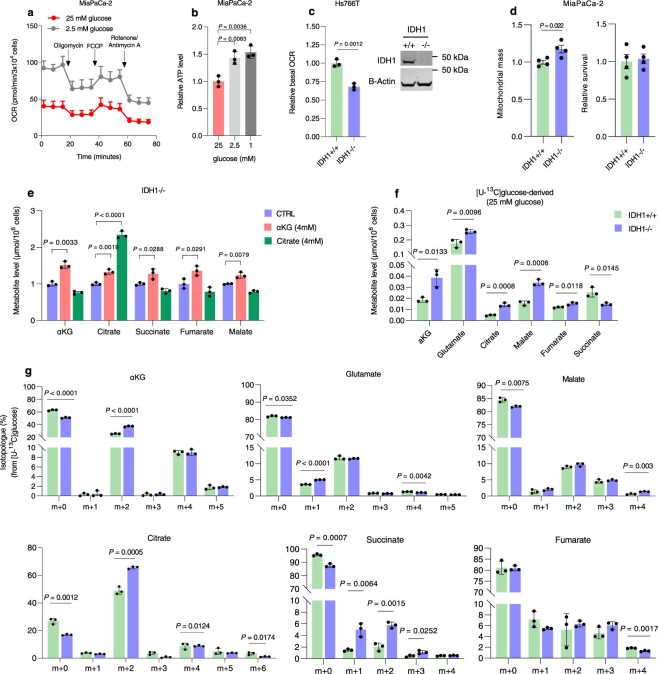
IDH1-/- cells have impaired mitochondrial function. **a**, Oxygen consumption rates (OCR) in MiaPaCa-2 cells cultured under the indicated glucose conditions for 30 hours (a representative of three independent biological replicates with similar results is shown). **b**, ATP levels in MiaPaCa-2 cells cultured under the indicated conditions for 24 hours (n = 3 independent biological replicates). **c**, Basal OCR in Hs766T pancreatic cancer cells cultured in 1 mM glucose for 24 hours (a representative of two independent biological replicates with similar results is shown). Western blot of IDH1 expression (representative immunoblots of two independent biological replicates with similar results are shown). **d**, Mitochondrial mass and cell viability in MiaPaCa-2 cells cultured with 2.5 mM glucose for 30 hours (n = 4 independent biological replicates). **e**, Relative abundance of the indicated TCA-related metabolites in MiaPaCa-2 cells cultured with unlabeled 2.5 mM glucose for 38 hours followed by unlabeled αKG (4 mM) or sodium citrate (4 mM) for 10 hours (n = 3 individual biological replicates). **f**, **g**, Total pool size (**f**) and isotopologue distribution (**g**) of indicated metabolites in cells cultured with unlabeled 25 mM glucose for 38 hours followed by 25 mM [U-^13^C]glucose for an additional 10 hours (n = 3 individual biological replicates). Data are provided as mean ± s.d. (**a**-**c,e**-**g**) or mean ± s.e.m. (**d**). Pairwise comparisons were conducted using two-tailed, unpaired Student’s *t*-tests. Source data

Alpha-ketoglutarate (the other product of IDH1-dependent oxidative decarboxylation; Fig. 1k) also plays an important role in the IDH1-mediated survival response, largely through anaplerotic support of mitochondria. Indeed, numerous studies revealed the reliance of cancer cells on mitochondria under nutrient limitation^19,20^. Consistent with these studies, parental PDAC cells (with normal levels of IDH1) adapted to glucose withdrawal by compensatorily increasing oxygen consumption and ATP production (Extended Data Fig. 2a,b). Metabolic profiling confirmed a reduction in αKG in IDH1^−/−^ cells under glucose withdrawal (Fig. 2a). Interestingly, there were no differences in oxygen consumption rate (OCR) between IDH1^−/−^ and IDH1^+/+^ PDAC cells under nutrient-replete conditions (Fig. 2b). However, mitochondria in IDH1-knockout cells failed to adapt sufficiently to low-glucose conditions across PDAC cell lines, underscoring the importance of this enzyme under glucose depletion (Fig. 2c and Extended Data Fig. 2c). Similarly, mitochondrial membrane potential analyses demonstrated improved polarization in IDH1-proficient cells with glucose limitation but impaired polarization in IDH1-knockout cells (Fig. 2d). Results were not attributable to a reduction in mitochondrial mass or to a reduction in cell viability during these experimental time intervals (Extended Data Fig. 2d). Exogenous αKG rescued mitochondrial OCR and mitochondrial membrane potential in IDH1^−/−^ cells (Fig. 2c,d), which illustrates retained potential functionality of mitochondria during the 2-day experiment and reliance on the IDH1 product (that is, αKG) for that function.

Mitochondrial dysfunction with IDH1 deficiency was further validated through metabolic profiling. Mitochondrial tricarboxylic acid (TCA) cycle-related metabolites were reduced in IDH1^−/−^ cells under low-glucose conditions, as measured by total pool metabolite abundance (all isotopomers) and metabolite-specific ^13^C-enrichment studies, respectively (Fig. 2e,f). These experiments suggest reduced carbon input from [U-^13^C]glucose into mitochondrial TCA metabolites in IDH1^−/−^ cells compared with IDH1^+/+^ cells. Other mitochondria-associated metabolites were also apparently reduced in IDH1^−/−^ cells, such as nucleotide triphosphates and derivatives (Fig. 2g). Exogenous αKG rescued metabolite levels of TCA metabolites in IDH1^−/−^ PDAC cells cultured under low-glucose conditions, further reaffirming the importance of this IDH1 product and again establishing retained functionality of mitochondria during the experiment. In contrast, citrate (positioned upstream of the cytosolic wtIDH1 reaction in the cytosol) failed to increase TCA metabolites in IDH1^−/−^ cells, presumably due to a requirement of IDH1 function (Extended Data Fig. 2e). Under high glucose levels, pooled and metabolite-specific ^13^C-enrichment studies did not reveal any substantial differences between IDH1^+/+^ and IDH1^−/−^ cells, showing yet again that the enzyme is extraneous under these favorable conditions (Extended Data Fig. 2f,g). Several studies have shown that mitochondrial dysfunction exacerbates oxidative stress within mitochondria^33–36^, since these are the greatest source of ROS. Consistent with this point, we observed an elevation in mitochondrial ROS levels in IDH1-deficient cells under low-glucose conditions (Fig. 2h), with near complete rescue by exogenous αKG (Fig. 2h). Coculture with this IDH1 metabolite partially restored cell viability under the same culture conditions (Fig. 2i).

**Extended Data Fig. 3:**
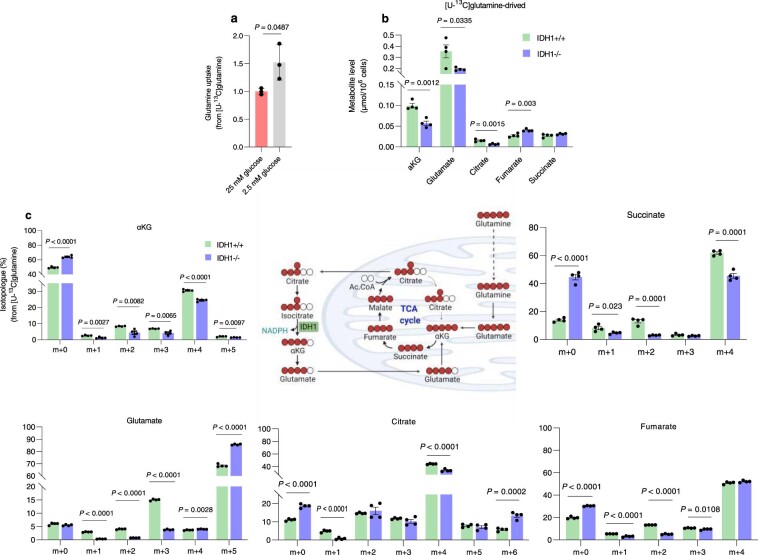
The impact of IDH1 on glutamine metabolism. **a**, Relative glutamine uptake was quantified via mass spectrometry in MiaPaCa-2 cells cultured with 4 mM [U-^13^C]glutamine under 25 or 2.5 mM glucose for 24 hours (n = 3 individual biological replicates). **b**, **c**, Total pool size (**b**) and isotopologue distribution (**c**) of indicated metabolites in cells cultured with unlabeled 2.5 mM glucose and 4 mM glutamine for 38 hours followed by 2.5 mM glucose and 4 mM [U-^13^C]glutamine for an additional 10 hours (n = 4 individual biological replicates). Data are provided as mean ± s.d. (**a**) or mean ± s.e.m. (**b**,**c**). Pairwise comparisons were conducted using two-tailed, unpaired Student’s *t*-tests. Source data

**Extended Data Fig. 4:**
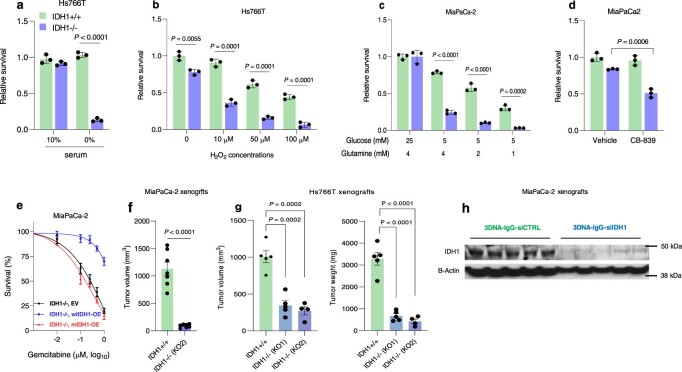
Impaired growth of IDH1-/- PDAC cells under cancer-associated stress. **a**, **b**, Relative survival as detected by Trypan blue assay in Hs766T cells under serum deprivation for 30 hours (**a**), or hydrogen peroxide and 2% serum for 72 hours (**b**) (n = 3 independent biological replicates). **c**, Relative survival of isogenic MiaPaCa-2 cells cultured in different concentrations of glucose and glutamine for five days (n = 3 independent biological replicates). **d**, Relative survival of isogenic MiaPaCa-2 cells cultured first in 2.5 mM glucose for 24 hours, followed by CB-839 (1 µM) treatment for an additional 24 hours (n = 3 independent biological replicates). **e**, Relative survival as detected by PicoGreen assay in IDH1-/- MiaPaCa-2 cells, transiently transfected with empty vector (EV), plasmid overexpressing catalytically active, or catalytically altered (R132H) IDH1, and treated with gemcitabine for 5 days under 5 mM glucose (relative glucose withdrawal from 25 mM baseline glucose) (n = 3 independent biological replicates). **f**, **g**, Growth of subcutaneous tumors from MiaPaCa-2 (**f**, n = 7 tumors per group) and Hs766T (**g**, IDH1 + / + , n = 5 tumors; IDH1-/- (KO1), n = 5 tumors; IDH1-/- (KO2), n = 4 tumors) in nude mice. **h**, Western blot analysis of IDH1 protein in xenografts treated with 3DNA-IgG-siCTRL or 3DNA-IgG-siIDH1 treated tumors shown in Fig. 3g (n = 5 tumors per group). Data are provided as mean ± s.d. (**a**-**e**) or mean ± s.e.m. (**f,g**). Pairwise comparisons were conducted using two-tailed, unpaired Student’s *t*-tests. Source data

Glutamine is a key upstream substrate for αKG through glutaminolysis, and this pathway is probably even more critical when glucose is scarce^37–40^. As expected, we observed increased glutamine uptake in parental PDAC cells under low-glucose conditions (Extended Data Fig. 3a). Since glutamine is not directly metabolized by IDH1, we did not anticipate a dramatic reduction of ^13^C-flux into the TCA cycle from [U-^13^C]glutamine in IDH1^−/−^ PDAC cells. Surprisingly, when IDH1-deficient PDAC cells were cultured under low-glucose conditions, a reduction in glutamine-derived carbon was in fact observed in several TCA-associated metabolites (Extended Data Fig. 3b,c). This is most probably attributable to a reduction in overall TCA cycling from impaired αKG anaplerosis. IDH1^−/−^ cells cultured under glucose limitation were also sensitive to additional oxidative insults, such as serum starvation, hydrogen peroxide, glutamine deprivation, glutaminase inhibitor CB-839 and chemotherapy (Extended Data Fig. 4a–e). In the latter experiment, stable re-expression of wtIDH1 (ref. ^7^) effectively rescued IDH1^−/−^ PDAC cells (that is, promoted chemotherapy resistance) while the catalytically altered mutant IDH1 did not. These data mirrored a previously published experiment by our group where stable reintroduction of the wtIDH1 isoenzyme in a heterozygous mIDH1 tumor had an unexpectedly greater impact on xenograft growth than restored expression of the mutant isoenzyme^30^.

### Loss of IDH1 impairs pancreatic tumor growth

**Fig. 3:**
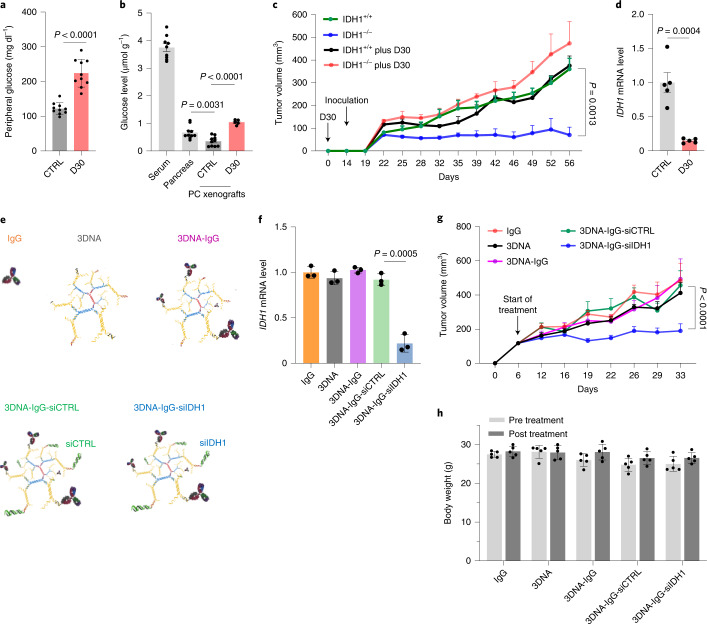
IDH1 is a therapeutic target in pancreatic cancer. **a**, Peripheral glucose levels in mice receiving normal water or D30 for 3 weeks (*n* = 10 mice per group). **b**, GC–MS analysis of intratumoral glucose levels (serum, *n* = 9 mice; pancreas, *n* = 10 mice; CTRL, *n* = 10 tumors; D30, *n* = 5 tumors). **c**, Growth of subcutaneous MiaPaCa-2 tumors in mice receiving normal water or D30 (*n* = 5 tumors per group; IDH1^+/+^ versus IDH1^–/–^ tumors, *P* = 0.0013). **d**, qPCR analysis of *IDH1* transcripts normalized to 18S in xenografts shown in **a** (*n* = 5 tumors per group). **e**, Cartoon depiction of 3DNA nanocarriers conjugated with IgG antibody (nonspecific targeting construct) and siRNAs. **f**–**h**, qPCR analysis of *IDH1* mRNA transcripts (**f**, *n* = 3 tumors per group), growth of subcutaneous tumors (**g**, *n* = 5 tumors per group; 3DNA-IgG-siCTRL versus 3DNA-IgG-siCTRL tumors, *P* < 0.0001) and body weights (**h**, *n* = 5 mice per group) from indicated treatment arms. Data provided as mean ± s.e.m. (**a**–**d**,**g**,**h**) or mean ± s.d. (**f**). Pairwise comparisons were conducted using two-tailed, unpaired Student’s *t*-tests. Longitudinal mixed models were fit for tumor growth, and time × treatment interactions were assessed. Source data

We speculated that proliferating tumors in vivo would be dependent on wtIDH1, since steep nutrient gradients are expected as tumors outgrow their blood supply^3,4,41,42^. Indeed, IDH1^−/−^ xenografts derived from two separate PDAC cell lines failed to proliferate compared with isogenic control xenografts (Extended Data Fig. 4f,g). Forced elevation of peripheral glucose levels through ad lib consumption of 30% dextrose water (D30 water) increased intratumoral glucose levels (Fig. 3a,b). Just as with in vitro studies (Fig. 1i), increased intratumoral glucose levels minimized the adverse impact of IDH1 deficiency on xenograft growth and reinforced the discovery that IDH1 is particularly critical to PDAC cells under low-glucose conditions (Fig. 3c). Interestingly, tumors in hyperglycemic mice had lower levels of *IDH1* expression (Fig. 3d), presumably related to a diminished dependence on the enzyme and mechanistically attributable to reduced cytoplasmic (‘activated’ form) HuR levels under these conditions^7^. Independent of these experiments, we silenced *IDH1* systemically in mouse tumors using 3DNA nanocarriers. 3DNA is a nanoscale, biodegradable DNA dendrimer with numerous single-stranded oligonucleotide extensions at the periphery of a globular 3DNA structure. These single-stranded oligos are available to hybridize customized effector molecules through complementary oligonucleotide linker moieties^43^. In the present study, 3DNA was derivatized with siRNAs targeting the *IDH1* transcript (Fig. 3e). Derivatized IgG served as a nonspecific ligand molecule that could potentially be replaced with cancer-specific ligands or antibodies for improved cancer cell targeting. When delivered systemically by intraperitoneal (i.p.) injection, the nanoparticle reduced *IDH1* mRNA and protein expression in subcutaneous xenografts in comparison with experimental control arms (Fig. 3f and Extended Data Fig. 4h). Just as we observed in xenograft studies with IDH1-knockout PDAC cells, systemic siIDH1 therapy slowed parental PDAC xenograft growth in mice (Fig. 3g). Mice showed no adverse physical effects of systemic siIDH1 therapy (Fig. 3h), consistent with a previous 3DNA study^43^ and separate studies of whole-body IDH1-knockout mice^44,45^.

### Reduced magnesium levels enhance potency of mutant IDH1 inhibitors against wild-type IDH1

**Fig. 4:**
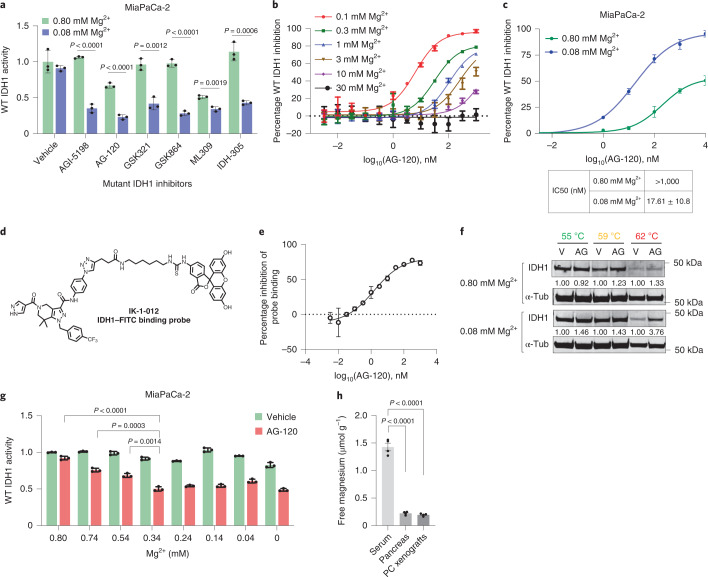
AG-120 inhibits wild-type IDH1 under low Mg^2+^ conditions. **a**, Wild-type (WT) IDH1 activity in MiaPaCa-2 pancreatic cancer cells cultured in medium containing 0.80 or 0.08 mM MgSO_4_ and 25 mM glucose for 24 h, followed by treatment with mIDH1 inhibitors (100 nM) for 6–8 h (one representative of three independent experiments with similar results is shown). **b**,**c**, Cell-free (**b**, *n* = 2 independent experiments) and cell-based (**c**, *n* = 3 independent biological replicates) IDH1 activity measurements following incubation with AG-120 under the indicated conditions. **d**,**e**, The structure of a newly synthesized IDH1 binding probe (**d**) and binding affinity of the probe in the presence of AG-120 (**e**, *n* = 2 independent experiments). **f**, Immunoblot analysis to evaluate thermal stability of the IDH1 protein in MiaPaCa-2 PDAC cells treated with vehicle (V) or AG-120 (AG, 1 µM) in medium containing 0.80 or 0.08 mM MgSO_4_ for 6–8 h (representative immunoblots of three biological replicates with similar results are shown). **g**, IDH1 activity of MiaPaCa-2 cells treated with AG-120 for 24 h under the indicated conditions (one representative of three independent biological replicates with similar results is shown). **h**, Free magnesium levels in normal pancreas, and MiaPaCa-2 pancreatic cancer xenografts versus serum (*n* = 4 samples per group). Data provided as mean ± s.d. (**a**–**c**,**e,g**) or mean ± s.e.m. (**h**). Pairwise comparisons were conducted using two-tailed, unpaired Student’s *t*-tests. Source data

**Extended Data Fig. 5:**
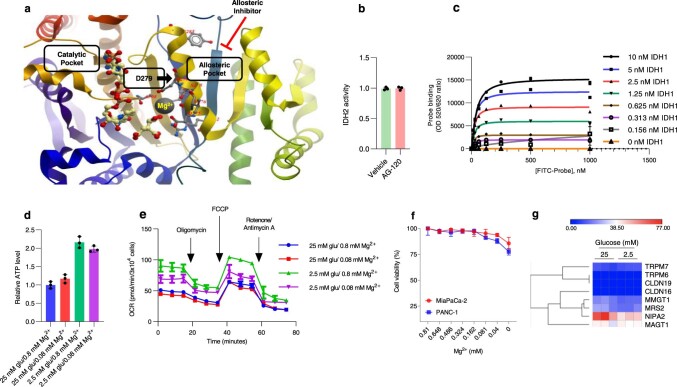
The impact of magnesium on pancreatic cancer cell metabolism and allosteric inhibitor binding to IDH1. **a**, Wild-type IDH1 from the Protein Database. Mg^2+^ is believed to interact with D279 (Asp^279^) in the allosteric pocket (accession number: PDB 1T0L). Under high Mg^2+^ conditions, the cation outcompetes the allosteric inhibitor, to render the drug ineffective. Under low Mg^+2^ conditions, the drug inhibits wtIDH1. **b**, The effect of AG-120 on IDH2 activity (cell-based assay) in MiaPaCa-2 PC cells (a representative of three independent biological replicates with similar results is shown. **c**, Validation of the fluorescent probe binding to wtIDH1 (n = 2 independent experiments). **d**, **e**, ATP levels (**d**, n = 3 independent biological replicates) and oxygen consumption rates (**e**, a representative of three independent biological replicates with similar results is shown) in MiaPaCa-2 cells under the indicated conditions. **f**, Cell viability of PDAC cells, cultured in MgSO_4_ at the indicated concentrations and 25 mM glucose for five days (n = 3 independent biological replicates). **g**, The impact of the indicated conditions and treatments on genes involved in magnesium homeostasis in MiaPaCa-2 cells (n = 3 individual biological replicates). Data are provided as mean ± s.d. (**b**-**f**). Pairwise comparisons were conducted using two-tailed, unpaired Student’s *t*-tests. Source data

Small molecule inhibitors of mIDH1 bind protein at an allosteric site remote from the catalytic pocket. Under normal conditions, Mg^2+^ interacts with Asp^279^ in the allosteric pocket to prevent inhibitor binding (Extended Data Fig. 5a)^46^. While both isoenzymes (mutant and wild type) possess this allosteric pocket, there is a widely held belief that Mg^2+^ outcompetes allosteric inhibitors for Asp^279^ in wtIDH1 due to a lower Km for Mg^2+^ as compared with the mutant isoenzyme (6,250 versus 19 µM, respectively)^46,47^. Thus, most allosteric inhibitors of IDH1 are believed to be highly selective for the mutant protein (with a higher Km for Mg^2+^) and only weak binders of wtIDH1 (ref. ^48^). Of note, there have been no rigorous analyses of the interaction between these compounds and wtIDH1 in cell-based studies under varying Mg^2+^ conditions, nor in in vivo cancer studies focused on conditions intrinsic to the TME. Consistent with previous studies^48^, conventional allosteric IDH1 inhibitors were generally ineffective against wtIDH1 under standard Mg^2+^ concentrations in cell culture (Fig. 4a). However, every single tested allosteric IDH1 inhibitor potently inhibited wtIDH1 activity at lower Mg^2+^ concentrations in cancer cells (Fig. 4a). This cell-based assay assessed on-target wtIDH1 activity through measurement of NADPH production from isocitrate and NADP^+^.

Ivosidenib was selected for downstream experiments since this drug performed well compared with other tested compounds (Fig. 4a), is well tolerated in patients and is already FDA approved for medically refractory IDH1-mutant acute myeloid leukemia and IDH1-mutant cholangiocarcinoma^49–51^. The drug was also highly potent in a cell-free wtIDH1 activity assay when Mg^2+^ concentrations were reduced to 0.1 mM (Fig. 4b). WtIDH1 activity was measured in a dose–response experiment with AG-120 in MiaPaCa-2 PDAC cells: half-maximal inhibitory concentration (IC_50_) after 24 h of incubation under both high (0.8 mM, similar to levels in serum or standard culture media) and low Mg^2+^ conditions (0.08 mM) was >1,000 and 17.6 nM, respectively (Fig. 4c). AG-120 had no inhibitory activity against the mitochondrial homolog wtIDH2 (Extended Data Fig. 5b). To confirm that AG-120 binds to wtIDH1 we generated a fluorescent probe, IK-1-012, which is structurally similar to an established IDH inhibitor with relatively poor selectivity (GSK321) for the mIDH1 isoenzyme^25,48,52^. We first determined that IK-1-012 binds to wtIDH1 with Kd = 87 nM, and then established a homogeneous time-resolved fluorescence (HTRF) binding assay for wtIDH1 (Fig. 4d,e and Extended Data Fig. 5c). Using this assay, we confirmed that AG-120 displaces the reference reporter IK-1-012 and binds to wtIDH1 with IC_50_ = 1.5 nM. A cellular thermal shift assay provided additional evidence that AG-120 and wtIDH1 interact (Fig. 4f). At higher temperatures, the compound stabilized the protein under low Mg^2+^ concentrations yet had no stabilizing effect at higher Mg^2+^ levels. In a cell-based wtIDH1 assay with variable Mg^2+^ levels, inhibition of wtIDH1 activity by AG-120 improved at lower Mg^2+^ concentrations and plateaued at <0.4 mM Mg^2+^ (Fig. 4g). Importantly, these lower Mg^2+^ concentrations are physiologically relevant because they simulate levels in the TME in the in vivo mouse model (Fig. 4h). These low Mg^2+^ levels are also consistent with a previous study showing reduced levels in human breast cancer tissues compared with serum^53^. Of note, low Mg^2+^ alone (absent the inhibitor) had a negligible effect on ATP production (Extended Data Fig. 5d), OCR (Extended Data Fig. 5e), cell viability (Extended Data Fig. 5f) or the expression of genes encoding proteins that regulate Mg^2+^ homeostasis (Extended Data Fig. 5g).

**Fig. 5:**
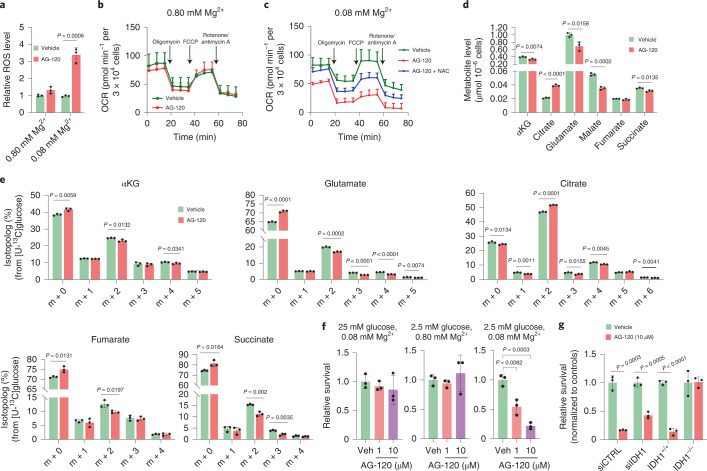
Low glucose and Mg^2+^ levels are required for anticancer activity by an allosteric IDH1 inhibitor (MiaPaCa-2 cells). **a**, ROS levels were detected by DCFDA assay in MiaPaCa-2 pancreatic cancer cells treated with AG-120 (1 µM) for 48 h under the indicated conditions and cultured in medium containing 2.5 mM glucose (*n* = 3 independent experiments). **b**,**c**, OCR in MiaPaCa-2 PDAC cells treated with vehicle or AG-120 cultured in medium containing either 0.80 mM Mg^2+^ and 2.5 mM glucose (**b**) or 0.08 mM Mg^2+^ and 2.5 mM glucose (**c**) for 30 h (representative experiments of three independent biological replicates with similar results are shown). **d**,**e**, Total pool size, including glucose-independent (m + 0) and glucose-dependent isotopologs (**d**) and isotopolog distribution (**e**) in cells cultured with unlabeled 2.5 mM glucose for 38 h followed by 2.5 mM [U-^13^C]glucose for an additional 10 h (*n* = 3 individual biological replicates). **f**, Relative clonogenic growth of cells treated with vehicle or AG-120 for 4 days under different levels of glucose and Mg^2+^ (*n* = 3 independent biological replicates). **g**, Relative clonogenic growth of indicated cells treated with vehicle or AG-120 for 4 days under 2.5 mM glucose and 0.08 mM Mg^2+^ (*n* = 3 independent biological replicates). Data provided as mean ± s.d. Pairwise comparisons were conducted using two-tailed, unpaired Student’s *t*-tests. Source data

**Extended Data Fig. 6:**
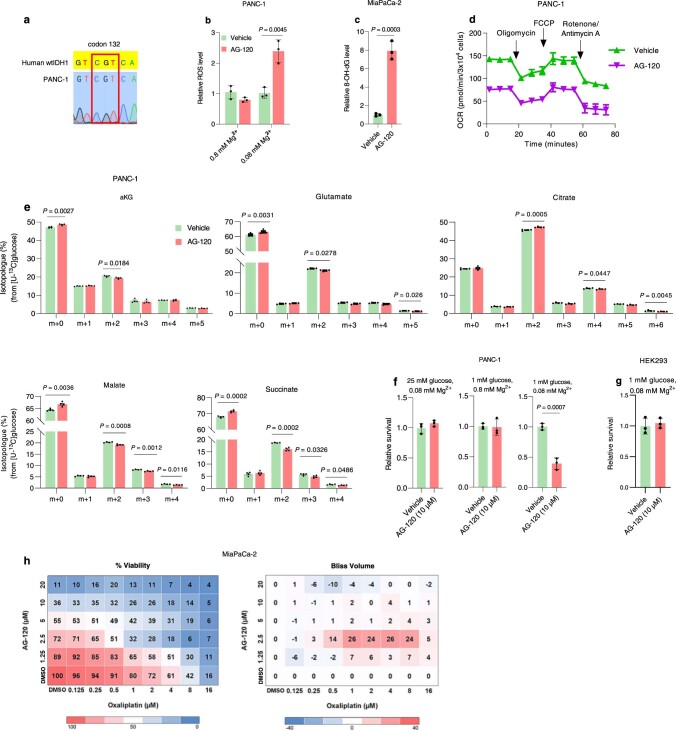
Effects of AG-120 against PDAC cells under low glucose and low Mg^2+^ conditions. **a**, Sanger sequencing of amplicons correlating with codon 132 of the wtIDH1 gene in PANC-1 pancreatic cancer cells. **b**, Total ROS levels detected in cells under the indicated treatments (n = 3 independent biological replicates). **c**, Oxidized DNA in MiaPaCa-2 cells as detected by 8-hydroxy-deoxyguanosine (8-OHdG) levels under 2.5 mM glucose and 0.08 mM Mg^2+^ for 48 hours (n = 3 independent biological replicates). **d**, Oxygen consumption rates PANC-1 cells treated with vehicle or AG-120 in media containing 0.08 mM Mg^2+^ and 2.5 mM glucose for 30 hours (a representative of three independent biological replicates with similar results is shown). **e**, Isotopologue distribution in PANC-1 cells cultured with unlabeled 2.5 mM glucose for 38 hours followed by incubation with 2.5 mM [U-^13^C]glucose for an additional 10 hours (n = 4 individual biological replicates). **f**, **g**, Relative clonogenic growth of PANC-1 (**f**, n = 3 independent biological replicates) and HEK293 (**g**, n = 3 independent biological replicates) cells treated with vehicle or AG-120 for five days under the indicated conditions. **h**, Percent viability and Bliss independence scores with a combination of AG-120 and oxaliplatin at the indicated doses in MiaPaCa-2 cells cultured in 2.5 mM glucose for five days (n = 3 independent biological replicates). Positive values reflect synergism between these two compounds in the cells. Data are provided as mean ± s.d. (**b**-**d**,**f**,**g**) or mean ± s.e.m. (**e**). Pairwise comparisons were conducted using two-tailed, unpaired Student’s *t*-tests. Source data

Allosteric IDH1 inhibitors exhibited substantial anticancer activity under low Mg^2+^ and metabolic conditions that require wtIDH1 function. For instance, AG-120 treatment induced a surge in intracellular ROS under low Mg^2+^ and low glucose in cultured wtIDH1 PDAC cells. This effect was not observed at higher Mg^2+^ levels (Fig. 5a and Extended Data Fig. 6a,b). Oxidative stress was even apparent within the nucleus of PDAC cells cultured under these conditions, which suggested a broad impact of IDH1 inhibition across subcellular compartments (Extended Data Fig. 6c). AG-120 phenocopied the metabolic effects of IDH1 knockout, suggesting on-target pharmacologic action against wtIDH1. The drug severely impaired oxygen consumption at reduced Mg^2+^ and glucose levels but had no effect on oxygen consumption when PDAC cells were also cultured under standard Mg^2+^ concentrations (Fig. 5b,c and Extended Data Fig. 6d). Furthermore, antioxidant supplementation partially rescued mitochondrial OCR in AG-120-treated cells (Fig. 5c). Under low glucose and Mg^2+^ concentrations, AG-120 reduced total pool metabolite abundance and enrichment of glucose-derived mitochondrial TCA isotopomers (Fig. 5d,e and Extended Data Fig. 6e). In these experiments, citrate (upstream from isocitrate) was largely increased (not decreased) with AG-120 treatment, consistent with blockade of wtIDH1. Human PDAC cells cultured under low Mg^2+^ and low glucose had markedly impaired survival with AG-120 exposure, yet no effect was observed when Mg^2+^ or glucose alone was reduced (Fig. 5f and Extended Data Fig. 6f). A noncancer cell line proved to be resistant to the drug under low Mg^2+^ and low glucose conditions, hinting at a potential therapeutic window (Extended Data Fig. 6g). siRNA silencing of IDH1 partially abrogated the effect of AG-120 under low Mg^2+^ and glucose, and IDH1^–/–^ cells were completely resistant to the drug (Fig. 5g), further validating on-target activity against wtIDH1. In vitro combination studies with a chemotherapeutic (oxaliplatin) suggest that wtIDH1 inhibition sensitizes cancer cells to a pro-oxidant and DNA-damaging agent. In this experiment the Bliss index model of AG-120 and oxaliplatin revealed a synergy between the two drugs, as reflected by a positive synergy score^54,55^. Several dosing combinations yielded Bliss indices >20 (Extended Data Fig. 6h).

**Extended Data Fig. 7:**
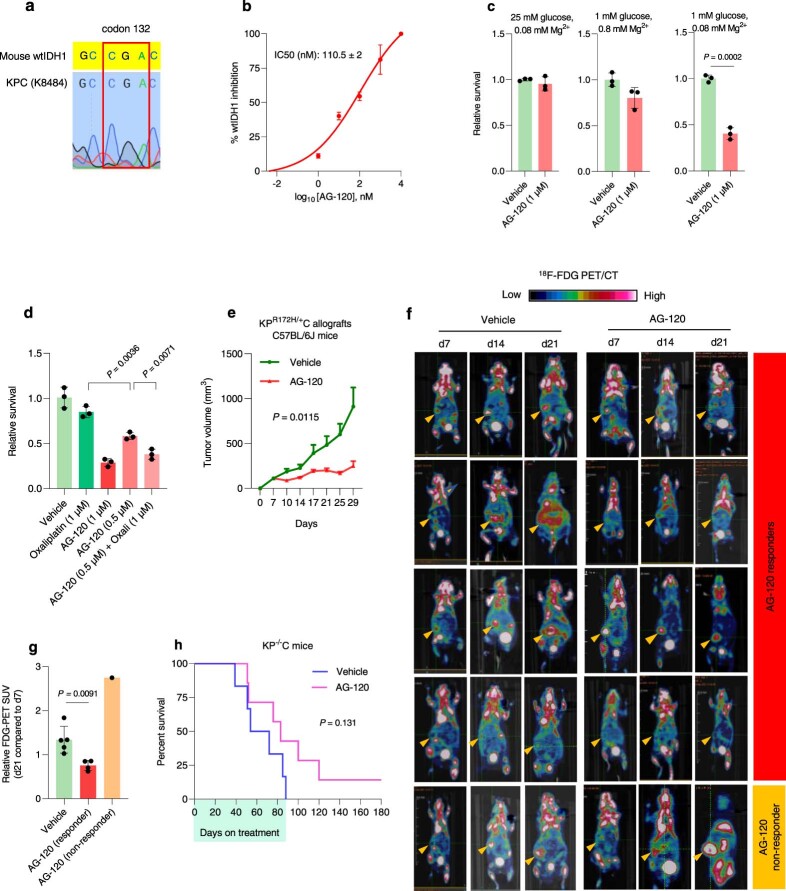
AG-120 activity against KPC cells. **a**, Sanger sequencing of amplicons correlating with codon 132 of the wtIDH1 gene in KPC murine pancreatic cancer cells (K8484). The reference murine wild-type sequence is shown. **b**, IDH1 activity of KPC cells treated with AG-120 for 24 hours in media containing 0.08 mM Mg^2+^ (n = 3 independent biological replicates). **c**, Relative clonogenic growth of KPC cells under the indicated conditions for 5 days (n = 3 independent biological replicates). **d**, Relative clonogenic growth of KPC cells with the indicated treatments, cultured with 1 mM glucose and 0.08 mM Mg^2^ for five days (n = 3 independent biological replicates). **e**, Growth of KPC subcutaneous xenografts in C57BL/6 J mice under the indicated treatment arms (vehicle, n = 5 tumors; AG-120, n = 4 tumors). **f, g**, CT/^18^F-FDG-PET images (**f**) and SUV values (**g**) of C57BL/6 J mice bearing orthotopic KPC tumors treated with vehicle or AG-120 (n = 5 mice per group). **h**, Survival analysis in tamoxifen-inducible KP^-/-^C (Kras^G12D/+^; Trp53^lox/lox^; Pdx1-Cre) mice treated with vehicle (n = 6 mice) or AG-120 (n = 7 mice). Data are provided as mean ± s.d. (**b**-**d**) or mean ± s.e.m. (**e**,**g**). Pairwise comparisons were conducted using two-tailed, unpaired Student’s *t*-tests. Longitudinal mixed models were fit for tumor size growth, and time by treatment interactions were assessed. Survival data are represented by Kaplan-Meier curves, and tests for treatment differences were conducted with Fleming-Harrington (0,1) weighted log rank test statistics. Source data

The amino acid sequence of IDH1 is conserved between human and mouse (95.7%), and the allosteric pocket is nearly identical. Wild-type IDH1 genetic sequence was confirmed in murine pancreatic cancer cell line KPC K8484 (Extended Data Fig. 7a). As with human PDAC, AG-120 reduced murine wtIDH1 activity and cell viability under low Mg^2+^ and low glucose (Extended Data Fig. 7b,c). Head-to-head under these conditions, the drug outperformed a standard-of-care chemotherapeutic (oxaliplatin) when given at the same dose (Extended Data Fig. 7d).

### PDAC is sensitive to allosteric IDH1 inhibition

**Fig. 6:**
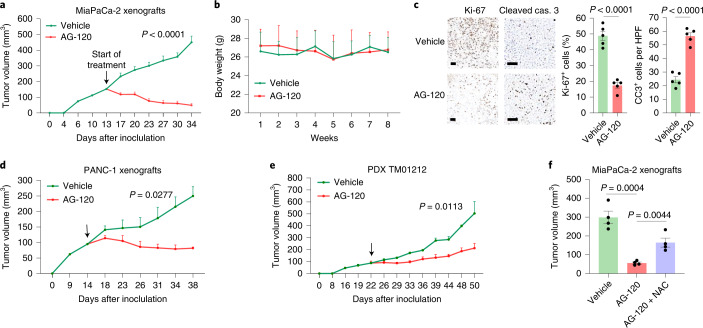
AG-120 inhibits pancreatic cancer growth in mice. **a**,**b**, For all animal experiments, mice were treated orally with vehicle or AG-120 (150 mg kg^−1^, twice daily) unless otherwise indicated. Start of treatment is denoted by an arrow. MiaPaCa-2 xenograft growth (**a**, *n* = 8 tumors per group) and body weight (**b**, *n* = 5 mice per group) of nude mice treated with vehicle or AG-120. **c**, Ki-67 and cleaved caspase 3 immunolabeling for tumors in **a**. **d**,**e**, Xenograft growth of PANC-1 (**d**, *n* = 5 tumors per group) and PDX TM01212 (**e**, 75 mg kg^–1^, once daily, i.p. administration; *n* = 4 tumors and *n* = *5* tumors for vehicle and AG-120, respectively). **f**, Independent MiaPaCa-2 xenograft experiment in nude mice (*n* = 4 tumors per group). Data provided as mean ± s.e.m. Pairwise comparisons were conducted using two-tailed, unpaired Student’s *t*-tests. Longitudinal mixed models were fit for tumor size growth, and time × treatment interactions were assessed. Source data

**Fig. 7:**
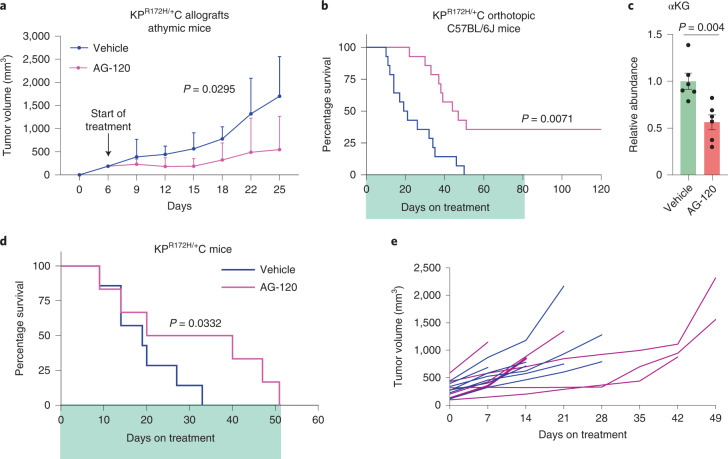
AG-120 activity in murine pancreatic cancer. **a**, Growth of subcutaneous allografts derived from murine pancreatic cancer (KPC K8484 cells) transplanted into nude mice. Mice were treated with vehicle or AG-120 (*n* = 3 tumors). **b**, Survival analysis of C57BL/6 J mice transplanted with orthotopic murine pancreatic cancer treated with vehicle or AG-120 (*n* = 14 mice per group). **c**, αKG levels in orthotopic murine pancreatic cancer treated with vehicle or AG-120 for 10 days (*n* = 6 tumors per group). **d**,**e**, Survival analysis (**d**) and tumor volume (**e**) measured by ultrasound in KPC (Kras^G12D/+^; Trp53^R172H/+^; Pdx1-Cre) mice treated with vehicle (*n* = 7 mice) or AG-120 (*n* = 6 mice). Data provided as mean ± s.d. (**a**) or mean ± s.e.m. (**c**). Pairwise comparisons were conducted using two-tailed, unpaired Student’s *t*-tests. Longitudinal mixed models were fit for tumor size growth, and time × treatment interactions were assessed. Survival data represented by Kaplan–Meier curves, and tests for treatment differences were conducted with Fleming–Harrington (0,1) weighted log-rank test statistics. Source data

For animal studies, AG-120 dosing schedules were selected (unless indicated) based on previous work testing the compound’s effect on mIDH1 tumors (150 mg kg^−1^ orally twice per day^56,57^). The drug resulted in flank xenograft shrinkage in vivo in two different PDAC cell lines, as well as in a patient-derived xenograft (PDX) model (Fig. 6a–e). Mouse weights were used as a surrogate marker of systemic toxicity, and were unchanged (Fig. 6b). Similar to in vitro studies (Fig. 1j and Extended Data Fig. 1h), the antioxidant NAC (1.2 g l^−1^ water) partially rescued the growth of AG-120-treated xenografts (Fig. 6f). In addition, AG-120 reduced the growth of subcutaneously implanted murine KPC allografts in nude mice (Fig. 7a). Syngeneic flank xenograft KPC tumors in C57BL/6 J mice were also responsive (Extended Data Fig. 7e). In a separate experiment on syngeneic tumors, mice bearing orthotopic KPC tumors transplanted into the pancreas by survival surgery experienced a twofold survival benefit with AG-120 treatment (19 versus 44 days). None of the vehicle-treated mice survived long term, while 35% of treated mice showed no clinical signs of cancer progression beyond 3 months (Fig. 7b). AG-120 drug activity was corroborated by CT/^18^F-FDG–positrom emission tomography (PET) functional imaging of syngeneic orthotopic tumors in a parallel experiment (Extended Data Fig. 7f,g). In another independent orthotopic study measuring metabolites, AG-120-treated tumors had reduced αKG levels (Fig. 7c). The effect of AG-120 was further examined in two separate autochthonous PDAC models. KP^R172H/+^C mice had improved survival with the study drug (performed in an independent laboratory by J.P.M.; Fig. 7d,e). A more aggressive strain revealed a trend towards improved survival (performed by A.V.-G.; Extended Data Fig. 7h), but did not reach statistical significance.

**Extended Data Fig. 8:**
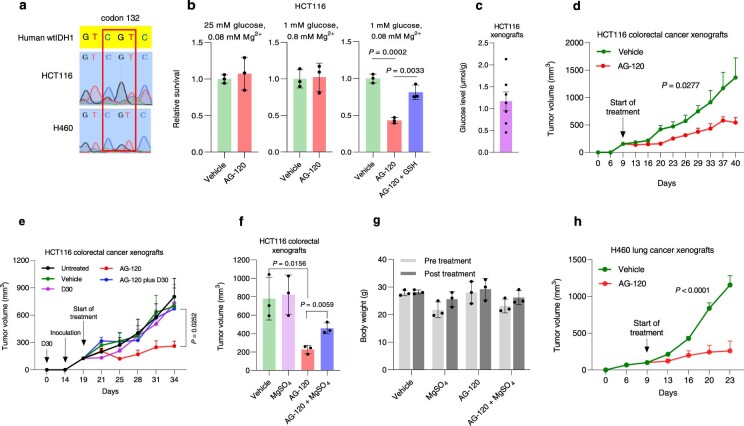
AG-120 activity in colorectal and lung cancer xenograft models. **a**, Sanger sequencing of codon 132 of the wtIDH1 gene in HCT116 colorectal and H460 lung cancer cells. **b**, Relative clonogenic growth of HCT116 cells treated with vehicle, AG-120 (10 µM), and GSH (4 mM) under the indicated concentrations of glucose and Mg^2+^ for five days (n = 3 independent biological replicates). **c**, Glucose levels in HCT116 colorectal xenografts harvested from nude mice (n = 7 tumors). **d**-**f**, Growth rate of HCT116 xenograft tumors (**d**, n = 4 tumors per group), and with forced hyperglycemia induced by D30 water consumption (**e**, n = 3 tumors per group; AG-120 vs AG-120 plus D30: *P* = 0.0252), or with water containing exogenous MgSO_4_ (**f**, n = 3 tumors per group). **g**, Body weights of nude mice bearing HCT116 xenografts treated as indicated (n = 3 mice per group). **h**, Relative growth of subcutaneous H460 lung xenografts in nude mice (n = 5 tumors per group). Data are provided as mean ± s.d. (**b**,**e**-**g**) or s.e.m. (**c**,**d**,**h**). Pairwise comparisons were conducted using two-tailed, unpaired Student’s *t*-test. Longitudinal mixed models were fit for tumor size growth, and time by treatment interactions were assessed. Source data

In a validation experiment using a separate tumor type, HCT116 colon cancer cells were also treated with AG-120 (Extended Data Fig. 8a). Similar to wtIDH1 PDAC cells, HCT116 cells were sensitive to AG-120 under low Mg^2+^ and glucose in vitro; exogenous reduced glutathione (GSH) mitigated the antitumor effects (Extended Data Fig. 8b). When injected into the flanks of nude mice, HCT116 xenografts also exhibited low intratumoral glucose levels (Extended Data Fig. 8c). Tumor proliferation was markedly reduced by oral AG-120 (Extended Data Fig. 8d,e). Ad libitum D30 (Extended Data Fig. 8e) and MgSO_4_ water (Extended Data Fig. 8f,g) both diminished the antitumor effects of AG-120 (that is, they rescued the tumors). Similarly, AG-120 inhibited the growth of a wtIDH1 lung cancer xenograft (Extended Data Fig. 8h).

**Fig. 8:**
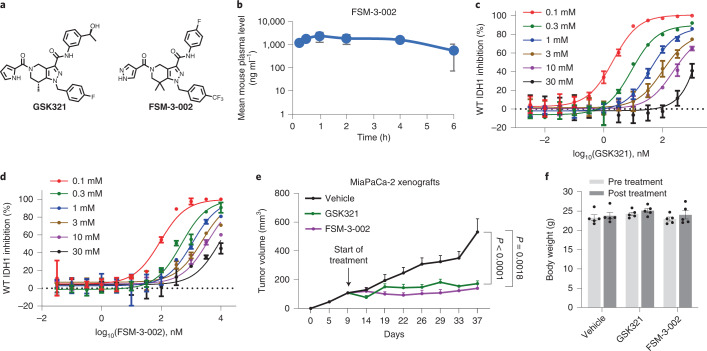
Alternative allosteric wild-type IDH1 inhibitors. **a**, Structures of GSK321 and FSM-3-002. **b**, PK analysis (*n* = 3 CD-1 mice per time point) for FSM-3-002 after 10 mg kg^−1^ i.p. administration. **c**,**d**, Cell-free analysis of wtIDH1 activity following incubation with GSK321 (**c**) or FSM-3-002 (**d**) under the indicated concentrations of Mg^2^ (*n* = 2 independent experiments). **e**. Growth of MiaPaCa-2 xenografts in mice injected i.p. with either vehicle, GSK321 (75 mg kg^−1^ once daily) or FSM-3-002 (75 mg kg^–1^ once daily) (*n* = 5 tumors per group). **f**, Body weights of nude mice bearing MiaPaCa-2 pancreatic cancer xenografts treated with either vehicle, GSK321 or FSM-3-002 (*n* = 5 mice per group). Data provided as mean ± s.d. (**b**–**d**) or mean ± s.e.m. (**e**,**f**). Pairwise comparisons were conducted using two-tailed, unpaired Student’s *t*-tests. Longitudinal mixed models were fit for tumor growth, and time × treatment interactions were assessed. For analyses in **e**, Bonferroni correction was employed so that a two-sided type I error level of 0.025 was adopted per test. Source data

Finally, anti-wtIDH1 effects of additional allosteric IDH inhibitors were more closely examined. Experiments included an available and previously published pharmacologic tool with modest wtIDH1 inhibitory activity (GSK321)^25,52^ and a derivative of this compound generated by our team (FSM-3-002) (Fig. 8a). FSM-3-002 was identified using a bioisosteric approach to replace the pyrrole of GSK321 with a more stable motif, resulting in superior metabolic stability. In a mouse liver microsome assay, the compound showed *t*_1/2_ = 147 min (Supplementary Note). Pharmacokinetic (PK) analysis in CD-1 mice was performed after i.p. administration at 10 mg kg^−1^, and plasma levels were measured at six time points (0.25, 0.5, 1, 2, 4 and 6 h). FSM-3-002 had good overall plasma exposure, with *C*_max_ = 2,325 ng ml^−1^ at 1 h (Fig. 8b). Both the known wtIDH1 inhibitor (GSK321) and FSM-3-002 were quite potent at low Mg^2+^ concentrations in a cell-free IDH1 activity assay (IC_50_ = 2.07 and 91.39 nM at 0.1 mM Mg^2+^ for GSK321 and FSM-3-002, respectively) (Fig. 8c,d). Both compounds were well tolerated in mice and caused a marked reduction in MiaPaCa-2 xenograft growth (Fig. 8e,f).

## Discussion

Wild-type IDH1 was identified as a potential therapeutic target in cancer a decade ago. Metallo et al. demonstrated that reductive carboxylation of glutamine-derived αKG (to isocitrate) by wtIDH1 supported lipogenesis in melanoma cells cultured under hypoxia^27^. The authors showed that suppression of the enzyme reduced cell growth. Mullen et al. demonstrated similar biochemistry under normoxia in cancer types with deficient mitochondria, including an osteosarcoma and a renal cell carcinoma cell line^26,58^. The same group also showed the importance of reductive carboxylation by wtIDH1 for spheroid growth in an in vitro lung cancer model^25^. More recently, Calvert et al. determined that the reverse wtIDH1 reaction, oxidative decarboxylation, supported glioma growth through the production of NADPH and that the oxidative IDH1 reaction served to control ROS in brain tumors^28^. Some of these previous studies employed less selective mIDH1 inhibitors (GSK321 and the related GSK864, which have mild wtIDH1 activity) as pharmacologic ‘tools’ to test the impact of wtIDH1 inhibition in vitro and, to a limited extent, in preclinical mouse models such as glioma^25,28,52^. Studies of whole-body wtIDH1 knockout revealed that wtIDH1 deletion did not affect mouse wellness at baseline, but mice were vulnerable to oxidative liver injury with sublethal doses of lipopolysaccharide^44,45^. This small, but illuminating, group of studies focused on wtIDH1 established several important principles: (1) the enzyme is a promising therapeutic target worthy of further study and (2) inhibition of the wtIDH1 target should have a reasonable safety profile against noncancer tissues.

The present study builds on these previous works by offering several insights. First, we show that nutrient limitation in the cancer microenvironment increases the importance of wtIDH1 for cancer cell survival while normal cells and tissues rely less on the enzyme, even under nutrient withdrawal. This was apparent not only in cell culture experiments, but also in mouse tissues where we observed minimal systemic toxicity. Certainly, the favorable safety profile of AG-120 in patients supports the probability of a promising therapeutic window^49,50^. Second, in numerous cell culture and animal experiments, wtIDH1 proved expendable to cancer cells under nutrient-replete conditions. When ambient glucose levels were increased, neither genetic deletion nor pharmacologic inhibition of wtIDH1 affected PDAC viability. These findings suggest that glycemic status could serve as a future biomarker of anti-wtIDH1 therapy and would further provide a rationale for tight glucose control in diabetic patients receiving treatment.

Third, the present work introduces the idea that the cytosolic enzyme, wtIDH1, is important for TCA cycle activity and other aspects of mitochondrial function. WtIDH1 activity is linked directly to mitochondrial function through generation of the anaplerotic substrate, αKG. Additionally, both αKG and NADPH produced by wtIDH1 help control ROS levels in the mitochondria, further supporting the organelle’s integrity. The data presented here also validate previous studies indicating the importance of wtIDH1 for redox homeostasis^25,26,28,58^. These mechanistic insights offer clues to potential combination therapies that could augment anticancer effects of AG-120. As an example, preliminary data presented here suggest that a combination of wtIDH1 and glutaminase inhibition may synergize by enhancing intratumoral ROS and impairing mitochondrial function. Additionally, chemotherapy is known to damage DNA through oxidative stress^59^ and the current study offers additional evidence that AG-120 is a promising therapeutic complement to chemotherapy.

Fourth, and most importantly, this study reveals that allosteric IDH1 inhibitors, which were designed to selectively target mIDH1, are even likely to be active against cancers that lack the mutant protein. Before this work, this possibility had been largely dismissed and insufficiently investigated. Herein we show that, under low ambient Mg^2+^ levels, wtIDH1 becomes susceptible to allosteric inhibition. Indeed, all tested, off-the-shelf allosteric inhibitors exhibited tremendous potency at nanomolar drug concentrations under low Mg^2+^ conditions. In the context of reduced Mg^2+^ and low nutrient levels present in the TME, cancer cells depend on wtIDH1 for antioxidant defense and mitochondrial function, and these drugs exhibit strong anticancer activity in this context. Thus, the FDA-approved mIDH1 inhibitor, AG-120, effectively treated numerous and diverse wtIDH1 cancers in mice. Strikingly, the drug substantially improved survival in both an orthotopic PDAC model and an autochthonous murine PDAC model.

Taken together, these data reveal that two principal conditions are required for AG-120 efficacy against wtIDH1 cancer: (1) reduced Mg^2+^ permits drug-inhibitory activity against wtIDH1 and (2) nutrient limitation (ubiquitous in the TME of PDAC) enforces cancer cell reliance on wtIDH1. Thus, tumors become vulnerable to wtIDH1 inhibition in preclinical models. This work provides a rationale for a pending clinical trial testing AG-120 in combination with chemotherapy to treat wtIDH1 PDAC. Additionally, optimization of anti-wtIDH1 therapy through rationale drug design and combination therapy is made possible with the mechanistic insights provided herein.

## Methods

### Cell lines, cell culture and reagents

All cell lines (MiaPaCa-2, PANC-1, Hs766T, H460, HCT116, HEK293) were obtained from the American Type Culture Collection (catalog nos. CRL-1420, CR-1469, HTB-134, HTB-177, CCL-247 and CRL-1573, respectively), except murine pancreatic cancer cells (KPC K8484: Kras^G12D/+^; Trp53^R172H/+^; Pdx1-Cre), which were provided by the Darren Carpizo laboratory^60^. Mycoplasma screening was performed using a MycoAlert detection kit (Lonza). Cell lines were maintained at 37 °C and 5% CO_2_. For standard cell culture, cells were grown in DMEM containing 25 mM glucose and 4 mM glutamine, supplemented with 10% FBS, 1% penicillin/streptomycin and prophylactic doses of plasmocin (Life Technologies, no. MPP-01-03) to prevent mycoplasma infection. Glucose withdrawal was performed to simulate low-glucose conditions in a pancreatic cancer microenvironment. For low-glucose experiments, glucose-free DMEM (Life Technologies, no. 21013-024) was supplemented with 2.5 mM glucose, 10% FBS and penicillin/streptomycin. For experiments with low magnesium and glucose, magnesium sulfate-depleted DMEM (Cell Culture Technologies, no. 964DME-0619) was supplemented with the indicated concentrations of MgSO_4_ and glucose. Standard or high Mg^2+^ refers to levels that approximate those in serum (0.8–1.5 mM); low levels approximate the tumor microenvironment and are generally <0.4 mM. For rescue experiments, sodium citrate dihydrate (Fisher Scientific, no. BP327), GSH (Sigma, no. G6013), αKG (Sigma-Aldrich, no. 75890), NAC (Sigma, no. A9165), wild-type IDH1 overexpression (Origene, no. SC116430) and mutant IDH1 (R132H) overexpression (Origene, no. RC400096) were used.

### CRISPR–Cas9 editing of IDH1 in pancreatic cancer cells

IDH1 knockout was performed using a guideRNA: GTAGATCCAATTCCACGTAGGG (Sigma, target ID no. HS0000323225). A negative control plasmid (CRISPR06-1EA) was used in isogenic cells. Plasmid transfections were performed with lipofectamine 2000 (Life Technologies, no. 11668-027). After 48 h, enhanced green fluorescent protein-expressing cells were sorted by flow cytometry. Clones from parental cell lines (MiaPaCa-2 and Hs766T) were expanded and genomic DNA and protein extracted for verification of IDH1 deletion. Herein, cells with IDH1 deletion and isogenic controls are referred to as IDH1^–/–^ and IDH1^+/+^, respectively.

### Cell viability assays

Cell viability was estimated through cell counting using Trypan blue (Life Technologies, no. 15250061) or by DNA quantitation via PicoGreen double-stranded DNA assay (Life Technologies, no. P7589). Drug combination efficacy was evaluated by the Bliss independence model as described previously^54,55^.

### Clonogenic assay

Cells (1,000–2,000 per well) were plated in six-well plates. Medium was not changed during experiments unless indicated. For AG-120 experiments, cells were first cultured with the indicated MgSO_4_ media for 24 h, followed by AG-120 (Cayman, no. 19894) treatment under 4 mM glutamine, 5% FBS and the indicated glucose concentrations. Upon completion of experiments, colonies were fixed in a reagent containing 80% methanol and stained with 0.5% crystal violet. To determine relative growth, dye was extracted from stained colonies with 10% acetic acid and the associated absorbance measured at 600 nm using a microplate reader (GloMax Explorer system, Promega).

### ROS and 8-OHdG quantification

Cells were incubated in a 96-well plate with 10 µM H2-DCFDA (Life Technologies, no. D399) for 45 min in serum-free media for detection of total intracellular ROS. For mitochondrial-specific ROS, cells were incubated with 5 µM MitoSOX Red (Life Technologies, no. M36008) for 30 min in serum-free medium. Cells were washed with PBS, and fluorescence was measured according to the manufacturer's instructions using a microplate reader (GloMax Explorer system, Promega). 8-OHdG was measured according to the manufacturer’s instructions (Abcam, no. ab201734). Readouts were normalized to cell number.

### RT–qPCR

Total RNA was extracted using PureLink RNA isolation (Life Technologies, no. 12183025) and treated with DNase (Life Technologies, no. AM2222). Complementary DNA was synthesized using 1 µg of total RNA, oligo-dT and MMLV HP reverse transcriptase (Applied Biosystems, no. 4387406). All PCR reactions were performed in triplicate using the following primers from Thermo Fisher Scientific: ME1 (4331182), ME2 (4331182), ME3 (4331182), IDH1 (4331182), IDH2 (4331182), MTHFD1 (4331182), MTHFD2 (4331182), G6PD (4331182), PGD (4331182), NNT (4331182), GLUD1 (4331182), GLUD2 (Bio-Rad, no. 457850923), ALDH3A1 (4331182), HuR (ELAVL1, 4331182) and 18S (4331182). Quantitative PCR with reverse transcription (RT–qPCR) acquisition was captured using Bio-Rad CFX96 and analyzed using Bio-Rad CFX Manager 2.0 software.

### RNA-seq and analyses

RNA quality was assessed via the Agilent 2100 Bioanalyzer (Agilent Technologies). A strand-specific RNA-seq library was prepared using the NEBNext Ultra II Directional RNA Library Prep Kit (NEB) according to the manufacturer’s protocols. RNA-seq was performed using the 150-base-pair, paired-end format on a NovaSeq 6000 (Illumina) sequencer. FastQC was used to assess RNA-seq quality and TrimGalore for adapter and quality trimming. RNA-seq reads were mapped against hg38 using the STAR (v.2.7.0e) aligner with default parameters. DESeq2 analysis with adjusted *P* < 0.05 was used to compile a list of differentially expressed genes.

### DNA-seq

DNA was isolated using the DNeasy blood and tissue kit (Qiagen) according to the manufacturer’s protocol. To assess wild-type IDH1 sequence, a portion of the IDH1 gene—exon 4 for human and exon 3 for murine cells, containing Arg132—was amplified using either of two pairs of primers (for human IDH1, F: 5'-ACCAAATGGCACCATACGA-3´; R: 5´-TTCATACCTTGCTTAATGGGTGT-3´; for mouse IDH1, F: 5´-ATTCTGGGTGGCACTGTCTT-3´; R: 5´-CTCTCTTAAGGGTGTAGATGCC-3´) and sequenced using one of the amplification primers. PCR reactions were generally carried out as follows: 95 °C for 30 s, 60 °C for 30 s and 72 °C for 40 s for a total of 40 cycles.

### Small RNA interference

For siRNA transfections, oligos were obtained from Ambion (Thermo Fisher Scientific) with the following ID numbers: ME1 (106787), ME2 (106789), ME3 (108082), IDH1 (107867), IDH2 (106706), G6PD (289142), PGD (112952), MTHFD1 (107902), MTHFD2 (108075), GLUD1 (107673), GLUD2 (121186), ALDH3A1 (106198), NNT (108343) and HuR (ELAVL1, 145882). Transfections were performed using Lipofectamine 2000. siRNA gene knockdown validation was determined 72 h after siRNA transfections via qPCR.

### Immunoblot analysis

Total protein was extracted with RIPA buffer (Pierce, no. 89900) supplemented with protease inhibitor (Life Technologies, no. 1861280) and quantified using the BCA Protein Assay (Thermo Fisher Scientific). Proteins were separated on 4–12% Bis-Tris gels (Life Technologies, no. MW04125) and transferred to polyvinylidene difluoride membranes. Membranes were probed with antibodies against IDH1 (Thermo Fisher Scientific, no. GT1521) overnight at 4 °C and alpha-tubulin (Invitrogen, no. 11224-1-AP) for 1–2 h at room temperature. Blots were probed with secondary antibodies customized for the Odyssey Imaging system (Thermo Fisher Scientific, no. 32106). The density of blots was quantified using Image Studio Software v.5.2.5.

### IDH activity assay

Cell-based wtIDH activity was measured by quantitation of IDH-dependent NADPH synthesis (Abcam, no. ab102528). This reaction measures the activity of both IDH1 and IDH2 isoenzymes. IDH1 activity was specifically measured using the same assay after transient siRNA silencing of IDH2, with the opposite approach to estimate IDH2 activity. All readouts were normalized to cell number. Cell-free wtIDH1 activity was performed by a fluorometric assay measuring NADPH levels at 355/460 nm. The assay contained 0.25 nM human recombinant IDH1 (RD systems), 30 µM isocitrate and 30 µM NADP^+^ in 20 µl of 100 mM Tris-HCL pH .08, 0.2 mM DTT, 0.05% CHAPS and MgCl_2_ at the indicated levels. Formation of NADPH was measured over 40 min at 5-min intervals.

### IDH binding assay

IDH1 binding was assessed using HTRF technology with 2.5 nM N-terminal HIS-tagged IDH1 (ActiveMotif) prebound to an anti-HIS terbium-conjugated antibody (PerkinElmer) and 200 nM 3',6'-dihydroxy-5-isothiocyanato-3H-spiro[isobenzofuran-1,9'-xanthen]-3-one (FITC)-labeled IDH1 probe, IK-1-012, in a final volume of 10 µl of IDH1 assay buffer (100 mM Tris-HCL pH 8.0, 0.2 mM DTT, 0.05% CHAPS and MgCl_2_ at the indicated levels). After an incubation time of 60 min at room temperature, time-resolved fluorescence at excitation of 340 nm and emission at 520 and 620 nm were measured using a ClarioStar plate reader (BMG Lab Tech) and the HTRF ratio at 520/620-nm emission values was calculated. Data are expressed as percentage inhibition, where 0 and 100% are equal to the HTRF ratio in the presence and absence, respectively, of HIS-IDH1.

### Cellular thermal shift assay

Thermal shift experiments were performed as previously described^61^. Briefly, cells were cultured in medium containing the indicated experimental concentrations of MgSO_4_ under standard conditions (25 mM glucose, 4 mM glutamine and 10% FBS) for 24 h. Cells were then treated with either vehicle (DMSO) or AG-120 (1 µM) for 6–8 h under the indicated Mg^2+^ levels. Cells were then trypsinized, washed with PBS, suspended in PBS (containing protease inhibitors), lysed through three freeze–thaw cycles in liquid nitrogen at 37 °C and then heated at the indicated temperatures for 3 min. Preparations were spun down at 13,000 r.p.m. for 10 min at 4 °C to remove denatured proteins, and supernatants were loaded on an immunoblot gel. Immunoblots were then performed as described.

### Cell bioenergetic assays

Oxygen consumption rates were measured using the Seahorse XFp Extracellular Flux Analyzer (Seahorse Bioscience) according to the manufacturer’s instructions. For OCR experiments, cells were cultured in 4 mM glutamine, 5% FBS and the indicated glucose levels for 24–36 h. Mitochondrial membrane potential was measured with tetramethyl rhodamine ethyl ester (TMRE) staining (Abcam, no. ab113852) according to the manufacturer’s instructions. Mitochondrial mass was measured with a mitochondrial marker, mitotracker green (Thermo Fisher Scientific, no. M7514), using two different approaches: a plate reader and live confocal imaging. NADPH (Abcam, no. ab65349), GSH/GSSG ratio (Promega, no. V6611) and ATP (Abcam, no. 83355) measurements were performed according to the manufacturer’s instructions. All readouts were normalized to cell number. For AG-120 experiments, cells were treated with AG-120 (2 µM) under 4 mM glutamine, 5% FBS and indicated glucose levels.

### Synthesis of FSM-3-002

The synthesis of FSM-3-002 is described in detail in the Supplementary Note, starting from commercially available tert-Butyl 5-(2-ethoxy-2-oxoacetyl)-3,3-dimethyl-4-oxopiperidine-1-carboxylate.

### Synthesis of IK-1-12

The synthetic scheme and characterization are provided in the Supplementary Note. In brief, N-(4-azidophenyl)-7,7-dimethyl-5-(1H-pyrazole-4-carbonyl)-1-(4-(trifluoromethyl) benzyl)-4,5,6,7-tetrahydro-1H-pyrazolo[4,3-c]pyridine-3-carboxamide was reacted with tert-butyl (6-(pent-4-ynamido)hexyl)carbamate in the presence of copper sulfate and sodium ascorbate in tert-butanol/water in a sealed tube, then heated in a microwave at 100 °C for 15 min to provide the triazole, tert-butyl (6-(3-(1-(4-(7,7-dimethyl-5-(1H-pyrazole-4-carbonyl)-1-(4-(trifluoromethyl)benzyl)-4,5,6,7-tetrahydro-1H-pyrazolo [4,3-c]pyridine-3-carboxamido)phenyl)-1H-1,2,3-triazol-4-yl)propanamido)hexyl)carbamate (IK-1-09), with 62% yield. This was then treated with 4 N HCl dioxane at 0 °C to room temperature for 1 h to remove the N-Boc protecting group, with 65% yield. The amine, IK-1-10, was then dissolved in dichloromethane and triethylamine (1.5 equivalents) and reacted with FITC (1 equivalent) to provide IK-1-12 after reverse-phase high-performance liquid chromatography purification (5–95% methanol in water), with 30% yield.

### Immunohistochemical staining

Samples were prepared with formalin and embedded in paraffin, followed by Ki-67 (Cell Signaling, no. 9027, rabbit monoclonal, 1:400 dilution) and cleaved caspase 3 (Asp175) (Cell Signaling, no. 9579, rabbit monoclonal, 1:250 dilution) immunolabeling.

### Magnesium measurement in murine tissues

Once animals were euthanized, tissues were collected, washed with 250 mM sucrose and quickly snap-frozen in liquid nitrogen. Tissues were homogenized in 10% sucrose via sonication on ice, followed by centrifugation at 12,000 r.p.m. for 10 min at 4°C. Supernatants were removed and examined for free Mg^2+^ content by atomic absorbance spectrophotometry (Agilent Technologies), adjusting for dilutions. Readouts were normalized according to homogenate volume and weight.

### Metabolic profiling

Experiments were performed in biological triplicate, and cells were grown in complete growth medium in T25 flasks. For metabolic profiling of IDH1^−/−^ and IDH1^+/+^ cells, processing began when cells reached 50% confluency. To prime cells for low-glucose conditions, standard culture medium was changed to low-glucose medium (2.5 mM glucose, 4 mM glutamine supplemented with 10% dialyzed FBS) for a 38-h incubation. Next, for metabolic flux profiling, cells were incubated in medium containing either 2.5 mM [U-^13^C]glucose (Cambridge Isotope Laboratories, no. CLM-1396-1) or 4 mM [U-^13^C]glutamine (Cambridge Isotope Laboratories, no. CLM-1822-H-0.1) for 10 h. For rescue experiments, once cells had completed 38 h of incubation under low (2.5 mM) glucose, cells were incubated with either unlabeled αKG (4 mM) or sodium citrate (4 mM) for 10 h.

Following incubation, cells were placed on ice, washed three times with cold PBS and lysed using ice-cold 80:20 methanol:water. For animal tissues and xenograft tumors, fragments weighing 15–25 mg were homogenized in ice-cold 80:20 methanol:water by sonication at 4 °C. Unlabeled γ-hydroxybutyrate (50 µM) was added to homogenized lysate as an internal standard. Cell extracts were centrifuged at 14,000 r.p.m. for 15 min at 4 °C. Supernatants were then dried using nitrogen gas. To prepare oxime derivatives, samples were incubated with 30 µl of freshly made MOX solution (40 mg methoxyamine hydrochloride in 1 ml of pyridine) for 45 min at 45°C. This was followed by incubation with 70 µl of MTBSTFA (N-(tert-butyldimethylsilyl)-N-methyltrifluoroacetamide) for 45 min at 45 °C to make silylated derivatives.

For glucose measurements, after sample drying with nitrogen gas, dried lysates were mixed with pyridine:acetic anhydride (1:2) solution and incubated at 60 °C for 30 min to convert glucose to its penta-acetate derivative. Samples were allowed to air dry at room temperature and then reconstituted in 100 µl of ethyl acetate. Results were normalized to m + 6 glucose as an internal standard.

Gas chromatography–mass spectrometery (GC–MS) analyses were performed using a Hewlett Packard 5973 Turbo Pump Mass Selective Detector and a Hewlett Packard 6980 Gas Chromatograph equipped with a DB-5ms GC Column (60 m × 0.25 mm × 0.25 µm, Agilent Technologies). Samples were injected in splitless mode. The column temperature was initially set at 100 °C and held for 1 min, then ramped by 8 °C min^−1^ to 170 °C and held for 5 min. Samples were then ramped at 5 °C min^−1^ to 200 °C and held for 5 min. Finally, samples were ramped at 10 °C min^−1^ to 300 °C and held 10 min. Masses were monitored in scan acquisition mode. Metabolomics data were analyzed using MSD ChemStation Software, v.F.01.03.2357 (Agilent Technologies). Metabolite counts were normalized using cell number from a parallel culture flask plated at an equivalent cell density. Isotopolog abundances were corrected for natural abundance using a correction matrix. Mass isotopomer distributions were computed as the ratio of mass isotopomer abundance divided by the sum of all mass isotopomer abundances for a particular metabolite, expressed as percentages. Metabolite pool size was determined using an internal standard and normalized to cell count.

Liquid chromatography–MS (LC–MS) analyses were performed using an Agilent Technologies Triple Quad 6470 LC–tandem MS (LC–MS/MS) system with a 1290 Infinity II LC Flexible Pump (Quaternary Pump), 1290 Infinity II Multisampler, 1290 Infinity II Multicolumn Thermostat with six port valves and a 6470 triple-quad mass spectrometer. Agilent MassHunter Workstation Software LC–MS Data Acquisition for 6400 Series Triple Quadrupole MS with v.B.08.02 was used for compound optimization and sample data acquisition. Agilent MassHunter Quantitative Analysis B.09.00 QqQ software was used to integrate and quantitate areas.

### In vivo studies

All experiments involving mice were approved by the CWRU Institutional Animal Care Regulations and Use Committee (IACUC, protocol no. 2018-0063). Mice were maintained under pathogen-free, temperature- and humidity-controlled conditions under a 12/12-h light/dark cycle and received normal chow (LabDiet, Prolab IsoPro RMH3000) in the animal facility. No additional food or nutrient-contained bedding was provided to the animals during these studies.

KPC (Kras^G12D/+^; Trp53^R172H/+^; Pdx1-Cre) mice were bred in-house at the CRUK Beatson Institute and maintained in conventional caging with environmental enrichment, as described previously^62^. Mice were fed ad libitum. Genotyping was performed by Transnetyx. Mice were monitored three times per week and, when a diagnosis of pancreatic cancer was made by abdominal palpation, this was confirmed by high-resolution ultrasound imaging using the VisualSonics Vevo 3100 preclinical imaging platform (FUJIFILM VisualSonics). Anesthesia was induced and maintained with a mixture of isoflurane and medical air. Mice were imaged weekly to monitor for tumor progression. Tumor volume was assessed for each mouse and plotted longitudinally. Mice of both sexes were recruited into the study and culled by the Schedule 1 method, as per Institutional guidelines when exhibiting moderate symptoms of PDAC (swollen abdomen, loss of body conditioning resembling cachexia, reduced mobility). All experiments with KPC mice were performed under a UK Home Office license and approved by the University of Glasgow Animal Welfare and Ethical Review Board. Tamoxifen-inducible KP^−/−^C (Kras^G12D/+^; Trp53^lox/lox^; Pdx1-Cre) mice were purchased from The Jackson Laboratory (no. 032429). Tamoxifen was dissolved in corn oil at 20 mg ml^−1^, and animals received tamoxifen (75 mg kg^−1^ i.p. once daily) for six consecutive days. Both genders were used in the study. Six-to-eight-week-old, female, athymic nude mice (Foxn1 nu/nu) were purchased from Harlan Laboratories (no. 6903 M) through ARC CWRU. PDX samples were purchased from The Jackson Laboratory (no. TM01212) and propagated in nude mice.

For flank xenograft experiments in mice, 1 × 10^6^ cells were suspended in 200 µl of a PBS:matrigel solution (1:1) and injected subcutaneously into the right flank. Treatment of flank tumors was initiated once these were palpable (120–150 mm^3^); maximal tumor size did not exceed 2,000 mm^3^ (size permitted by our institutional animal protocol). For orthotopic experiments, 4 × 10^4^ luciferase-expressing KPC K8484 cells were suspended in 30 µl of a PBS:matrigel solution (1:1) and injected into the pancreas of C57BL/6 mice at 12 weeks of age. Equal numbers of male and female mice were used. Mice were anesthetized using isoflurane gas. After ensuring the appropriate depth of anesthesia, a 0.5-cm left subcostal incision was made to gain access to the peritoneal cavity. The tail of the pancreas was externalized and the mixture carefully injected into the pancreas. The pancreatic tail was not handled for 1 min, to limit tumor cell leakage, and the organ was then returned to the peritoneal cavity. The incision was then closed with a combination of permanent suture and skin clips. On postoperative days 6–7 the presence of pancreatic tumors was confirmed by bioluminescence imaging (IVIS) using 100 µl i.p. of luciferin injection (50 mg ml^−1^). Mice with confirmed tumors were then randomized to the indicated treatment arms.

For the indicated experiments, hyperglycemia was induced by allowing mice to drink D30 water starting 2 weeks before cancer cell implantation. 3DNA nanocarriers were prepared as described previously^63^.

For 3DNA experiments, reagents (containing 0.0625 µg µl^−1^ 3DNA, 0.057 µg µl^−1^ IgG and 0.758 µM siRNA) were diluted in PBS and injected i.p. every 3 days.

For the indicated experiments, AG-120 (Asta Tech, no. 40817) was suspended in a vehicle containing 10% PEG-400, 4% Tween-80 and 86% saline for maximal dissolution of the drug. The solution was kept in 4 °C during these studies. The AG-120-containing cocktail was administrated twice daily at 150 mg kg^−1^ with at least 8 h between doses, unless otherwise indicated. An equal volume of vehicle was administrated to control mice. GSK321 (MedKoo Biosciences, no. 407271) and FSM-3-002 (Salvino Lab, Wistar Institute) were administrated daily at 75 mg kg^−1^. NAC (1.2 g l^−1^ water) and MgSO_4_ (1.5 g l^−1^ water) were used for the indicated rescue experiments.

For all flank xenograft experiments, tumor volumes were measured twice per week using a caliper (volume = length × width^2^/2). Upon termination of animal experiments, mice were euthanized using carbon dioxide inhalation followed by cervical dislocation, and tumors were harvested. Body weights were measured once weekly for the indicated times.

For ^18^F-FDG PET/CT Imaging, mice were fasted 5 h before ^18^F-FDG injection but had access to water. Subsequently, they were injected with ^18^F-FDG (150–200 µCi per animal). For dynamic scans, mice were scanned for 90 min; for static scans, a 30-min scan was performed 40 min after ^18^F-FDG injection. Quantitative image analysis of tumor ^18^F-FDG uptake was performed using Carimas software. Radioactivity data were decay corrected and normalized to the body weight of the mice and the amount of ^18^F-FDG injected. Tumor radioactivity concentration is expressed in terms of standardized uptake value as a function of time.

Mouse liver microsome stability data were obtained from Alliance Pharma, Inc. A general procedure for evaluation of metabolic stability in mouse liver microsomes is provided in brief. The test compounds and positive controls, at a final concentration of 0.5 μM, were incubated with 0.5 mg ml^−1^ liver microsomes and an NADPH-regenerating system (cofactor solution) in potassium phosphate buffer (pH 7.4). At 0, 5, 15, 30 and 45 min, an aliquot was taken and reactions were quenched with a solution of acetonitrile containing an internal standard. Additionally, controls not containing the cofactor solution were measured. Following completion of the experiment, samples were analyzed by LC–MS/MS. Results are reported as peak area ratios of each analyte to internal standard. Intrinsic clearance was determined from the first-order elimination constant by nonlinear regression.

Mouse PK study data were obtained from Alliance Pharma, Inc. In brief, a one-arm PK study was conducted in 18 CD-1 mice (*n* = 3 mice per time point). The animals were administered an i.p. dose of test compound at 10 mg kg^−1^. Dose solutions were formulated in 10% DMSO/10% Solutol/80% PBS. Plasma samples were collected at 0.25, 0.5, 1, 2, 4 and 6 h post dose. Following the PK study, samples were analyzed by LC–MS/MS and calculated concentrations of the test compound are reported.

### Statistics and reproducibility

No statistical methods were used to predetermine sample sizes, but our sample sizes are similar to those reported in previous studies^7,64^. No data were excluded from the analyses. The investigators were not blinded to allocation during experiments and outcome assessment. The treatments used in the study made it difficult to blind. While no formal randomization techniques were used, animals were allocated randomly to the control and experimental arms. Additionally, in vitro experiments were assigned randomly to control and experimental groups in different plates (that is, six- or 96-well plates) and formats (that is, at different positions in the plate), and were performed by different investigators for a number of independent replicates to prevent experimental bias. We often purchase reagents from different vendors to evaluate the reproducibility of our outcomes. Further information on research design is available in the Nature Research Reporting Summary linked to this article.

Data are provided as mean ± s.d. from three independent experiments. We made the recommended adjustment to show s.e.m. when more than three experiments were performed. Unless stated otherwise, two-sided type I error levels of 0.05 are assumed for hypothesis tests. For pairwise comparisons, *P* values were calculated using two-tailed unpaired Student’s *t*-tests using GraphPad Prism 9. For longitudinal models of tumor size, we use mixed models with random intercept and autoregressive order 1 serial covariance. We consider days from implantation as a categorical variable, and focus on the day × treatment interaction to assess whether there are significantly different trajectories of tumor size by treatment arm. Two-sided *P* values are reported that correspond to the test for interaction effects. When multiple treatment arms are considered, *P* values are obtained from pairwise two-treatment models. Survival data are represented by Kaplan–Meier curves, and tests for treatment differences are conducted with Fleming–Harrington (0,1) weighted log-rank test statistics^65^.

### Reporting summary

Further information on research design is available in the Nature Research Reporting Summary linked to this article.

## Supplementary information

Supplementary Information FSM-3-002 synthesis, MLM stability and IK-1-12 scheme.

Reporting Summary

## Data Availability

Open source software were used for RNA-seq analysis: FastQC (https://www.bioinformatics.babraham.ac.uk/projects/fastqc/), Trim Galore (http://www.bioinformatics.babraham.ac.uk/projects/trim_galore/), R (v.3.6.3 and v.3.4.2), STAR (v.2.7.0e), DESeq2 (v.1.26.0) and RSEM (v.1.3.2). RNA-seq data were deposited in Gene Expression Omnibus with accession no. GSE161073. DNA sequences were deposited in GenBank with accession nos. ON022872–ON022876. Metabolomics have been deposited in the EMBL-EBI MetaboLights database with accession no. MTBLS4722. Source data for Figs. 1–8 and Extended Data Fig. 1–8 are provided as Source Data files. All other data supporting the findings of this study are available from the corresponding author on reasonable request. Source data are provided with this paper.

## Source data

Source Data Fig. 1 Statistical Source Data.
Source Data Fig. 1 Uncropped blots.
Source Data Fig. 2 Statistical Source Data.
Source Data Fig. 3 Statistical Source Data.
Source Data Fig. 4 Statistical Source Data.
Source Data Fig. 4 Uncropped blots.
Source Data Fig. 5 Statistical Source Data.
Source Data Fig. 6 Statistical Source Data.
Source Data Fig. 7 Statistical Source Data.
Source Data Fig. 8 Statistical Source Data.
Source Data Extended Data Fig. 1 Statistical Source Data.
Source Data Extended Data Fig. 2 Statistical Source Data.
Source Data Extended Data Fig. 2 Uncropped blots.
Source Data Extended Data Fig. 3 Statistical Source Data.
Source Data Extended Data Fig. 4 Statistical Source Data.
Source Data Extended Data Fig. 4 Uncropped blots.
Source Data Extended Data Fig. 5 Statistical Source Data.
Source Data Extended Data Fig. 6 Statistical Source Data.
Source Data Extended Data Fig. 7 Statistical Source Data.
Source Data Extended Data Fig. 8 Statistical Source Data.
