# Supplementary material for: Limited nutrient availability in the tumor microenvironment renders pancreatic tumors sensitive to allosteric IDH1 inhibitors
Source: Nat Cancer. 2022 Jun 9;3(7):852–65. doi: 10.1038/s43018-022-00393-y (PMC9325670; doi:10.1038/s43018-022-00393-y)

nature portfolio

https://doi.org/10.1038/s43018-022-00393-y

Supplementary information

Limited nutrient availability in the tumor microenvironment renders pancreatic tumors sensitive to allosteric IDH1 inhibitors

In the format provided by the authors and unedited

Synthetic Scheme, Experimental Protocols and Spectral Characterization for FSM-3-002

Intrinsic Clearance of FSM-3-002 and Midazolam Control in Mouse Liver Microsomes

Synthetic Scheme and Spectra Characterization for IDH1 Binding Probe IK-1-12

Synthetic route of FSM-3-002

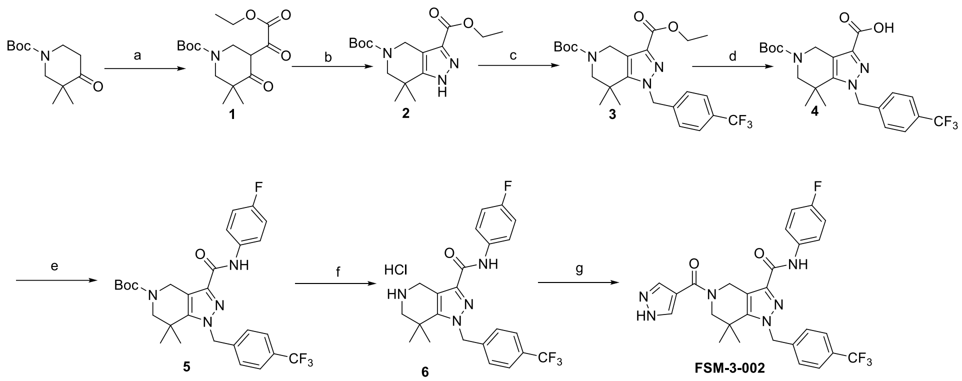

Reagents and conditions: (a) Diethyl oxalate, LDA, anhydrous THF, -78 ºC-r.t; 8h (b) hydrazine hydrate, Acetic acid, rt, 8h ; (c) 1-(Bromomethyl)-4-(trifluoromethyl)benzene, NaH, anhydrous THF, 0 ºC-rt, 8h; (d) NaOH, Ethanol:H_2O (3:1), rt; 4h; (e) 4-Fluoroaniline, HATU, DIPEA, DMF, rt, 1h; (f) 4N HCl in dioxane, dcm, 0 º C-rt, 2h; (g) 1*H*-Pyrazole-4-carboxylic acid, EDCI, HOBt, DIPEA, THF, rt, 6h.

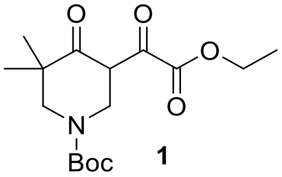

1

FSM-1-105

***tert*-Butyl 5-(2-ethoxy-2-oxoacetyl)-3,3-dimethyl-4-oxopiperidine-1-carboxylate**: To a stirred solution of *tert*-butyl 3,3-dimethyl-4-oxopiperidine-1-carboxylate (20 g, 88 mmol) in anhydrous THF (250 mL) was added LDA (2.0 M THF 50.6 mL, 101.2 mmol) at -78 °C dropwise. The reaction mixture was stirred at -78 °C for one hour and a solution of diethyl oxalate (15.57 g, 106.48 mmol) in anhydrous THF (50 mL) was added. The resulting reaction mixture was warmed to room temperature and stirred overnight. After completion of the reaction as indicated by TLC, water (500 mL) was added. The aqueous phase was neutralized with 1 N HCl and extracted with ethyl acetate (4 x 150 mL). The organic layer was washed with brine (500 mL), dried over Na_2SO_4, concentrated under reduced pressure and the crude residue was purified by flash chromatography using (0-20 % ethyl acetate in hexanes). Roto evaporation yielded the pure compound **1**(16.97 g, 51.9 mmol, 51.8 % yield) as a yellow oil. ^{1}H NMR (400 MHz, $CDCl_3$) δ 4.46 (s, 2H), 4.36 (d, J = 6.9 Hz, 2H), 3.40 (s, 1H), 1.59 (s, 2H), 1.49 (d, J = 9.9 Hz, 9H), 1.39 (t, J = 7.0 Hz, 3H), 1.21 (s, 6H). **MS (ESI):** *m/z* 350.32 $[M+23]^+$.

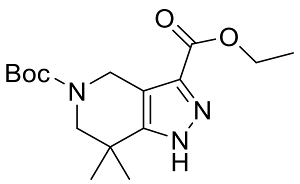

2

FSM-5-20

5-*tert*-Butyl 3-ethyl 7,7-dimethyl-6,7-dihydro-1*H*-pyrazolo[4,3-*c*]pyridine-3,5(4*H*)-dicarboxylate: To a solution of *tert*-butyl 5-(2-ethoxy-2-oxoacetyl)-3,3-dimethyl-4-oxopiperidine-1-carboxylate (16.97 g, 51.9 mmol) in acetic acid (40 mL) was added hydrazine hydrate (4 mL, 124 mmol) portion wise at room temperature. The mixture was stirred at room temperature overnight then poured into ice cold saturated aqueous sodium bicarbonate and extracted with ethyl acetate (3 x 150 mL). The combined organic layer was washed with brine (300 mL) and dried over Na_2SO_4 and evaporated under vacuum. The crude residue was purified by flash chromatography using (0-25 % ethyl acetate in hexanes) to provide the desired compound **2** (14.6 g, 46.4 mmol, 89.3% yield) as a white solid. ^{1}H NMR (400 MHz, $CDCl_3$) δ 4.66 (d, *J* = 17.8 Hz, 2H), 4.37 (d, *J* = 6.3 Hz, 2H), 3.42 (s, 2H), 1.50 (s, 9H), 1.38 (s, 3H), 1.29 (d, *J* = 10.2 Hz, 6H). **MS (ESI):** *m/z* 324.22 $[M+1]^+$.

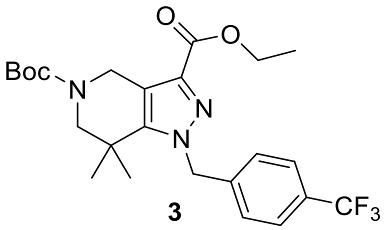

3

VY-9-108

5-*tert*-Butyl 3-ethyl 7,7-dimethyl-1-(4-(trifluoromethyl)benzyl)-6,7-dihydro-1*H*-pyrazolo[4,3-*c*]pyridine-3,5(4*H*)-dicarboxylate: To a solution of 5-*tert*-butyl 3-ethyl 7,7-dimethyl-6,7-dihydro-1*H*-pyrazolo[4,3-*c*]pyridine-3,5(4*H*)-dicarboxylate (10.94 g, 33.9 mmol) in anhydrous THF (150 mL) was slowly added 60 % sodium hydride (1.63 g, 40.6 mmol) at room temperature. After 1 hour, a solution of 1-(bromomethyl)-4-trifluoromethylbenzene (6.72 g, 35.6 mmol) in anhydrous THF (5 mL) was added and the mixture was stirred at room temperature overnight. The reaction mixture was quenched with water (100 mL) and the aqueous layer was extracted with ethyl acetate (3 x 100 mL). The combined organic layer was washed with brine (200 mL), dried over sodium sulfate, filtered, and concentrated under reduced pressure. The crude residue was purified by flash chromatography using (0-25 % ethyl acetate in hexanes) to provide

the desired compound **3** (7.2 g, 16.8 mmol, 50.0 % yield) as a yellow oil. ^{1}H NMR (400 MHz, $CDCl_3$) δ 7.58 (d, *J* = 8.2 Hz, 22H), 7.16 (d, *J* = 3.4 Hz, 2H), 5.55 (s, 2H), 4.67 (d, *J* = 21.3 Hz, 2H), 4.12 (q, *J* = 7.1 Hz, 2H), 3.39 (s, 2H), 1.48 (s, 9H), 1.17 (s, 6H). **MS (ESI):** *m/z* 482.25 $[M+1]^+$.

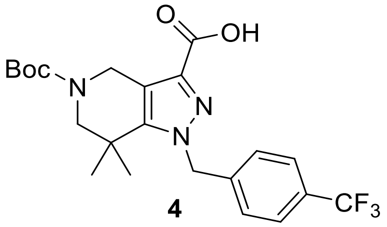

4

VY-9-097

5-(*tert*-butoxycarbonyl)-7,7-dimethyl-1-(4-(trifluoromethyl)benzyl)-4,5,6,7-tetrahydro-1*H*-pyrazolo[4,3-*c*]pyridine-3-carboxylic acid: To a solution of 5-*tert*-butyl 3-ethyl 7,7-dimethyl-1-(4-(trifluoromethyl)benzyl)-6,7-dihydro-1*H*-pyrazolo[4,3-*c*]pyridine-3,5(4*H*)-dicarboxylate (4.3 g, 10 mmol) in EtOH (40 mL) was added sodium hydroxide (0.8 g, 20 mmol) in water (20 mL). The resulting mixture was stirred at room temperature for 4 hours, concentrated under reduced pressure, diluted with water (40 mL) and washed with ethyl acetate (100 mL). The pH of the aqueous layer was adjusted to 6 with 1 N HCl and the resulting precipitate was collected by filtration and dried to give desired compound **4** (3.03 g, 7.52 mmol, 75 % yield) as a white solid. VY-10-097: ^{1}H NMR (400 MHz, DMSO) δ 7.70 (d, *J* = 8.2 Hz, 2H), 7.25 (d, *J* = 7.8 Hz, 2H), 5.58 (s, 2H), 4.51 (s, 2H), 3.30 (s, 2H), 1.41 (s, 9H), 1.11 (s, 6H **MS (ESI):** *m/z* 454.25 $[M+1]^+$.

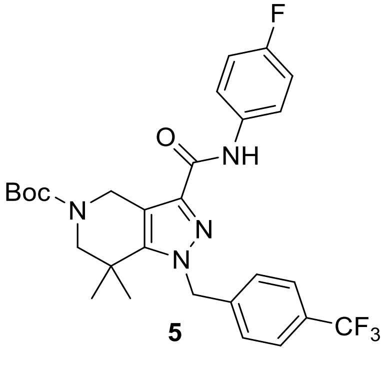

5

VY-10-261

***tert*-butyl 3-(4-fluorophenylcarbamoyl)-7,7-dimethyl-1-(4-(trifluoromethyl)benzyl)-6,7-dihydro-1*H*-pyrazolo[4,3-*c*]pyridine-5(4*H*)-carboxylate:** To a stirred solution of 5-(*tert*-butoxycarbonyl)-7,7-dimethyl-1-(4-(trifluoromethyl)benzyl)-4,5,6,7-tetrahydro-1*H*-pyrazolo[4,3-*c*]pyridine-3-carboxylic acid (0.95 g, 2.11 mmol) in anhydrous DMF (10 mL) was

added HATU (1.60 g, 4.22 mmol) and after stirring the mixture for 5 min DIPEA (0.98 g, 7.59 mmol) was added. The reaction mixture was then stirred for an additional 10-15 min. Then 4-fluoroaniline (0.26 g, 2.32 mmol) was added. The reaction mixture was stirred at room temperature for an additional 2 hours. After completion of the reaction, as monitored by tlc, water was added to the reaction mixture, and the aqueous layer was extracted with ethyl acetate (3 x 50 mL). The combined organic layer was washed with brine, dried over sodium sulfate, and evaporated under vacuum. The crude residue was purified by flash chromatography using (0-50% EA in hexanes) to obtain the pure compound (1.01 g, 88.01 % yield) as a white solid. ^{1}H NMR (400 MHz, $CDCl_3$) δ 8.56 (s, 1H), 7.65 – 7.57 (m, 4H), 7.14 (d, *J* = 7.3 Hz, 2H), 7.03 (t, *J* = 8.6 Hz, 2H), 5.51 (s, 2H), 4.75 (s, 2H), 3.41 (s, 2H), 1.48 (s, 9H), 1.20 (s, 6H).**MS (ESI):** *m/z* 569.28 $[M+23]^+$.

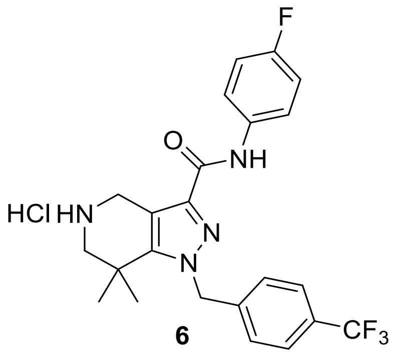

6

VY-10-262

***N*-(4-fluorophenyl)-7,7-dimethyl-1-(4-(trifluoromethyl) benzyl)-4,5,6,7-tetrahydro-1*H*-pyrazolo[4,3-*c*]pyridine-3-carboxamide hydrochloride(6):** To a solution of compound **4,** *tert*-butyl 3-(4-fluorophenylcarbamoyl)-7,7-dimethyl-1-(4-(trifluoromethyl)benzyl)-6,7-dihydro-1*H*-pyrazolo[4,3-*c*]pyridine-5(4*H*)-carboxylate (1.01 g, 1.856 mmol) in 10 ml dichloromethane, was added 10 ml of 4N HCl in dioxane slowly at 0 ^{O}C. The mixture was stirred at room temperature for 2-3h. after starting material disappeared reaction mixture concentrated under vacuum, toluene was added to precipitated white solid several times and evaporated under vacuum to remove traces of dioxane, and finally formed solid was filtered, washed with 10% ethyl acetate/hexane, and dried over vacuum to obtain desired compound **6** (890 mg, 99.5%) as HCl salt. ^{1}H NMR (400 MHz, DMSO) δ 10.32 (s, 1H), 9.66 (s, 2H), 7.85 – 7.78 (m, 2H), 7.75 (d, *J* = 8.2 Hz, 2H), 7.33 (d, *J* = 8.1 Hz, 2H), 7.25 – 7.05 (m, 2H), 5.73 (s, 2H), 4.31 (s, 2H), 3.21 (s, 2H), 1.35 (s, 6H). **MS (ESI):** *m/z* 447.34 $[M+1]^+$.

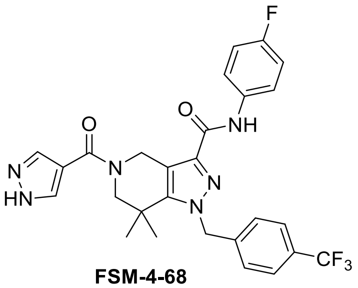

FSM-4-68
FSM-3-002

***N*-(4-Fluorophenyl)-7,7-dimethyl-5-(1*H*-pyrazole-4-carbonyl)-1-(4-(trifluoromethyl)benzyl)-4,5,6,7-tetrahydro-1*H*-pyrazolo[4,3-*c*]pyridine-3-carboxamide**: To a stirred solution of 1*H*-pyrazole-4-carboxylic acid (0.212 g, 1.90 mmol) in anhydrous DMF (10 mL) was added EDCI (0.327 g, 2.85mmol) and after stirring the mixture for 5 min, HOBt (384 mg, 2.85mmol), DIPEA (0.575 g, 5.7 mmol) was added. The reaction mixture was then stirred for an additional 10-15 min. Then *N*-(4-fluorophenyl)-7,7-dimethyl-1-(4-(trifluoromethyl)benzyl)-4,5,6,7-tetrahydro-1*H*-pyrazolo[4,3-c]pyridine-3-carboxamide hydrochloride (0.890 mg, 1.84 mmol) was added. The reaction mixture was stirred at room temperature for an additional 8 hours. After completion of the reaction, as monitored by tlc, water was added to the reaction mixture, and the aqueous layer was extracted with ethyl acetate (3 x 50 mL). The combined organic layer was washed with brine, dried over sodium sulfate, and evaporated under vacuum. The crude residue was purified by flash chromatography using (0-90 % EA in hexanes) to obtain the pure compound (780mg**,** 78.2 % yield) as white solid. ^{1}H NMR (400 MHz, $CDCl_3$) δ 8.53 (s, 1H), 7.97 (s, 2H), 7.62 (d, *J* = 8.2 Hz, 2H), 7.57 (dd, *J* = 8.8, 4.7 Hz, 2H), 7.16 (d, *J* = 8.2 Hz, 2H), 7.04 (t, *J* = 8.7 Hz, 2H), 5.52 (s, 2H), 5.09 (s, 2H), 3.75 (s, 2H), 1.28 (s, 6H). **^{13}C NMR** (101 MHz, DMSO) δ 164.22, 160.85, 159.90, 157.52, 147.04, 142.85, 141.09, 139.66, 135.37, 135.34, 130.60, 128.98, 128.67, 128.35, 128.04, 127.58, 126.01, 125.98, 125.94, 123.30, 122.79, 122.71, 120.60, 116.40, 115.69, 115.61, 115.39, 54.13, 34.08, 25.73, 25.55, 25.13. (^{19}F splitting present). **MS (ESI):** *m/z* 541.27 $[M+1]^+$.

^{1}H NMR and LC-MS of compound 1

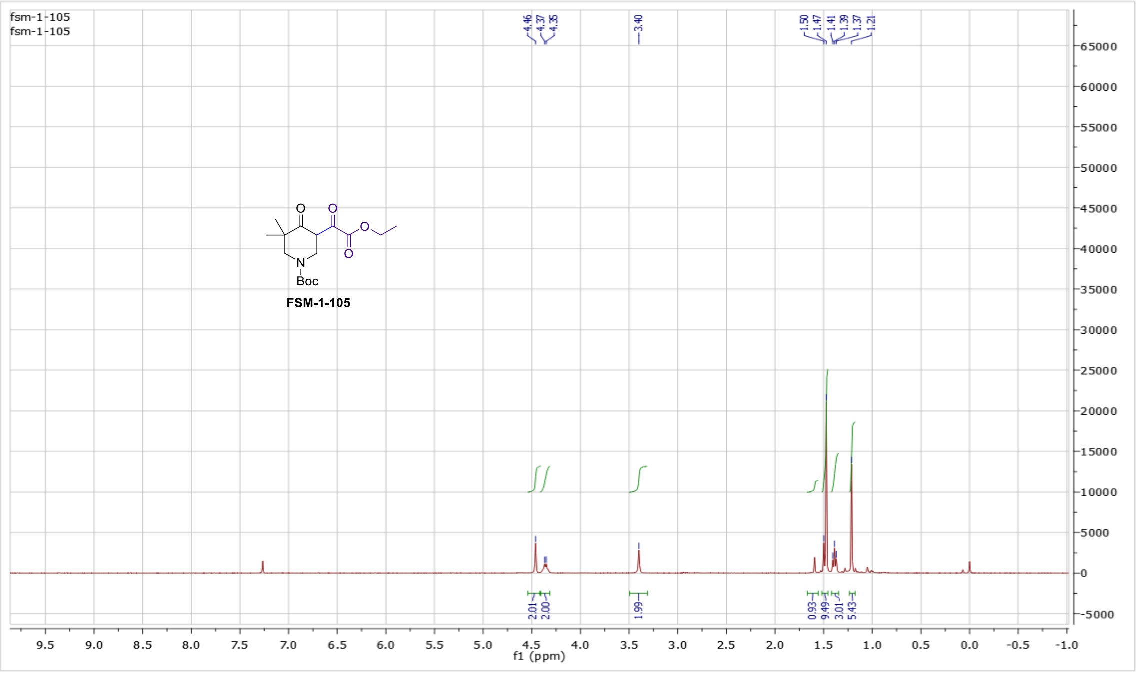

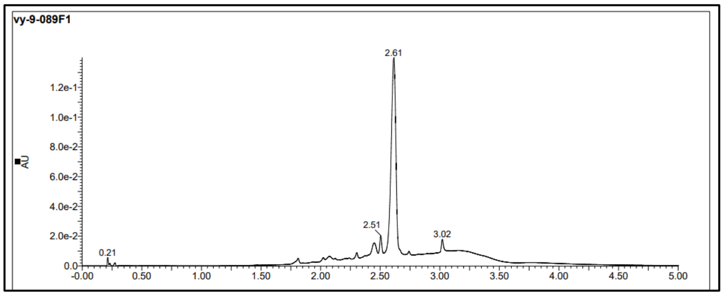

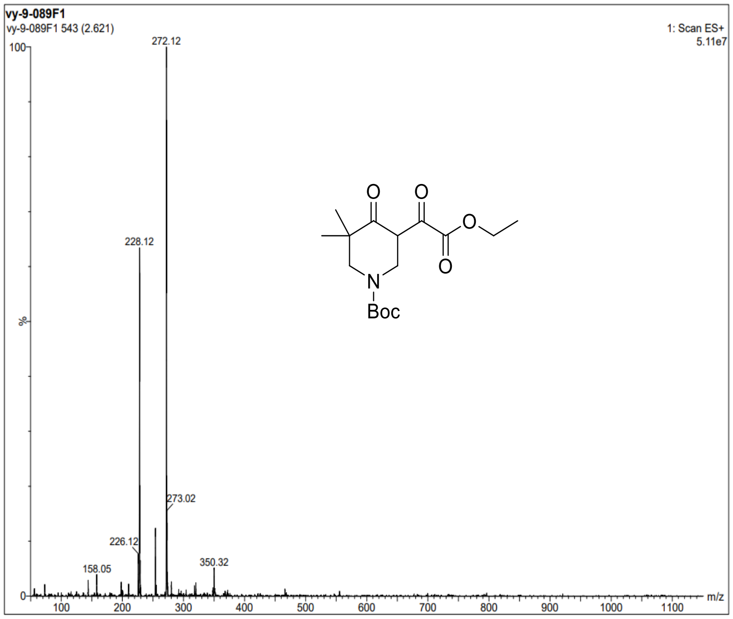

^{1}H NMR and LC-MS of compound 2

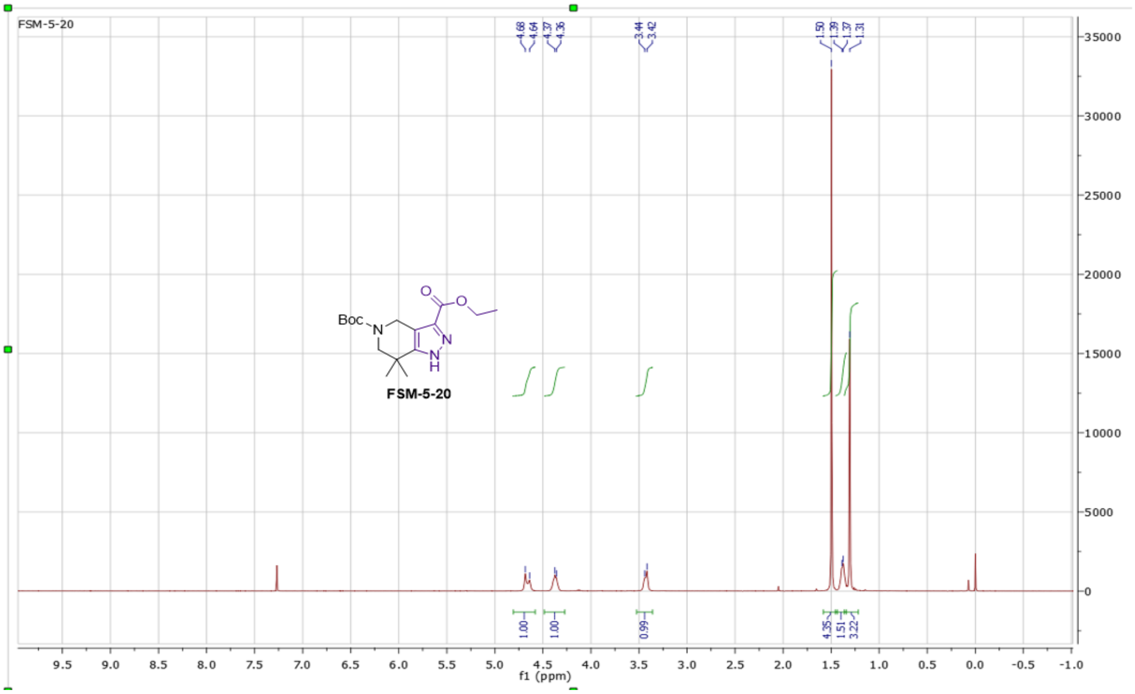

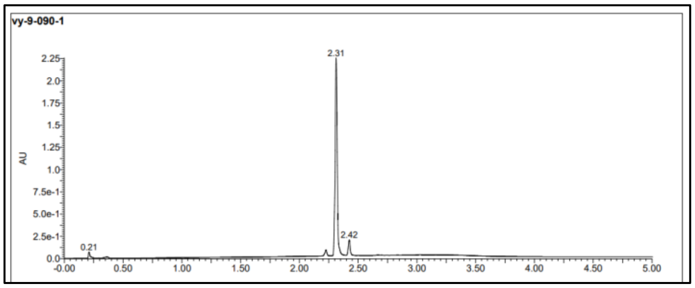

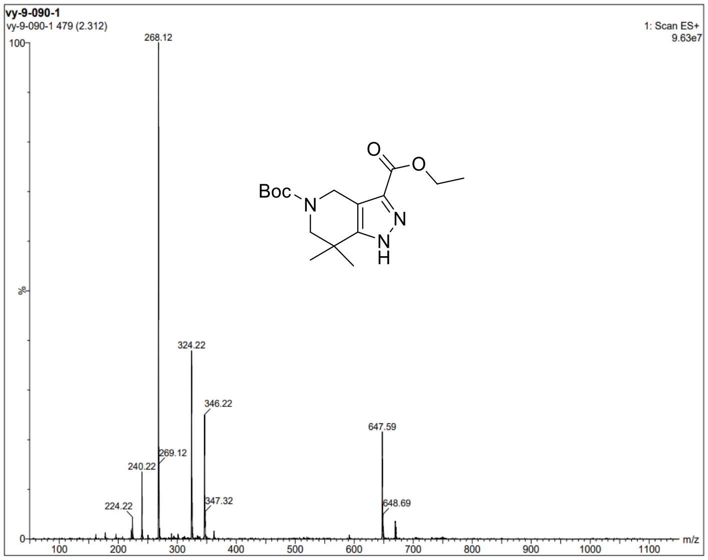

^{1}H NMR and LC-MS of compound 3

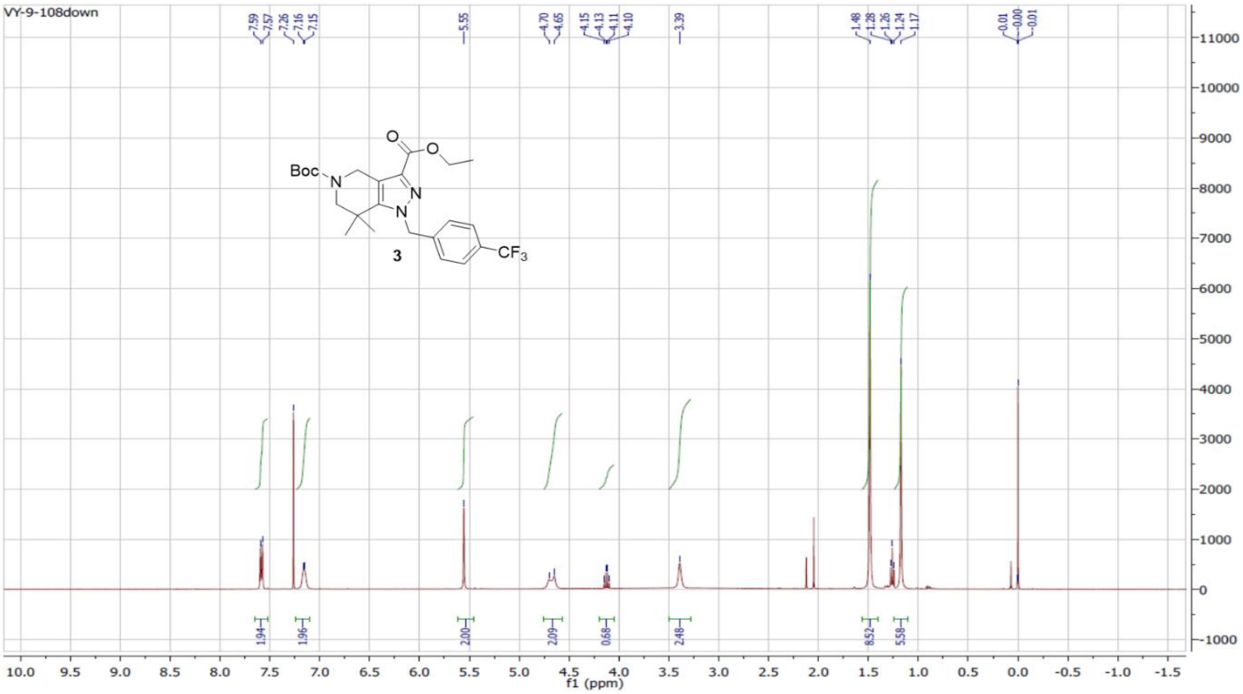

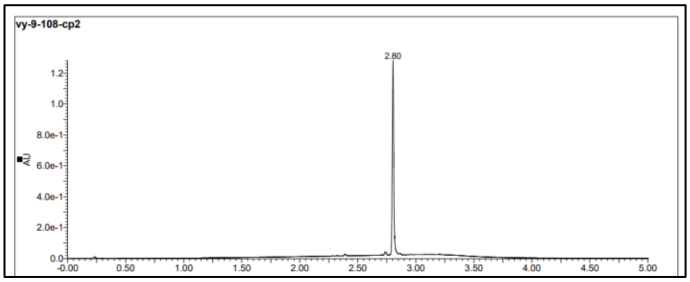

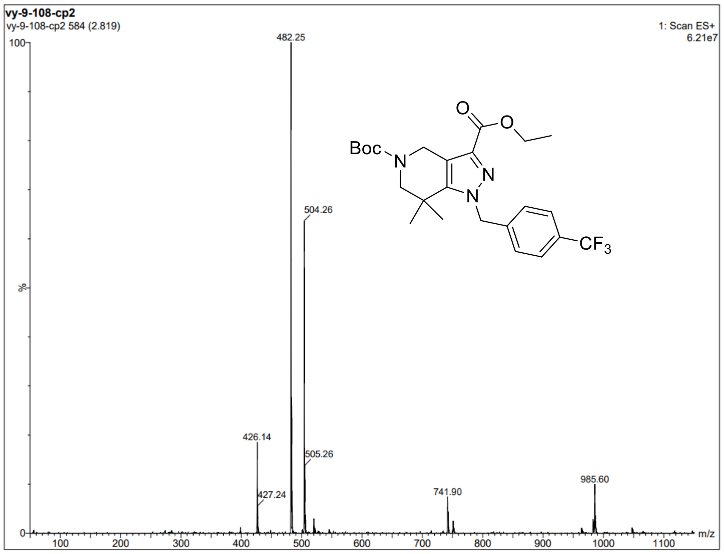

^{1}H NMR and LC-MS of compound 4

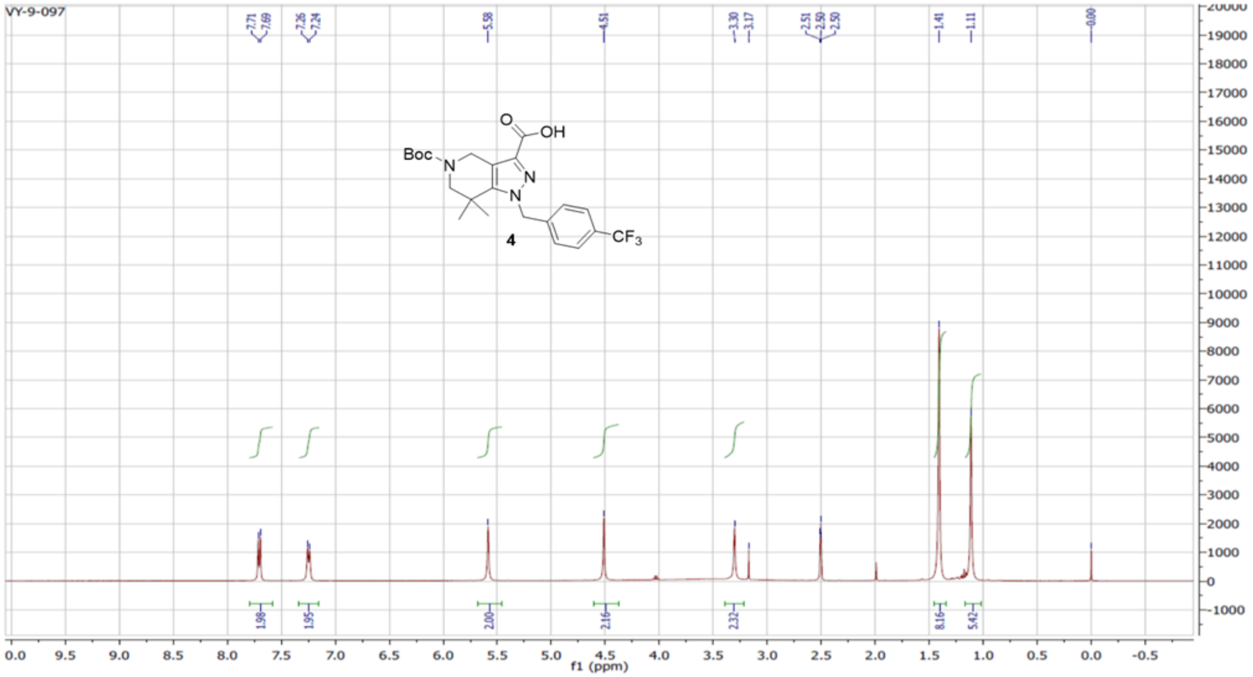

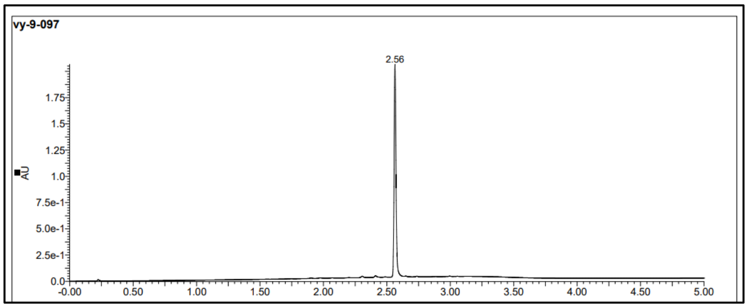

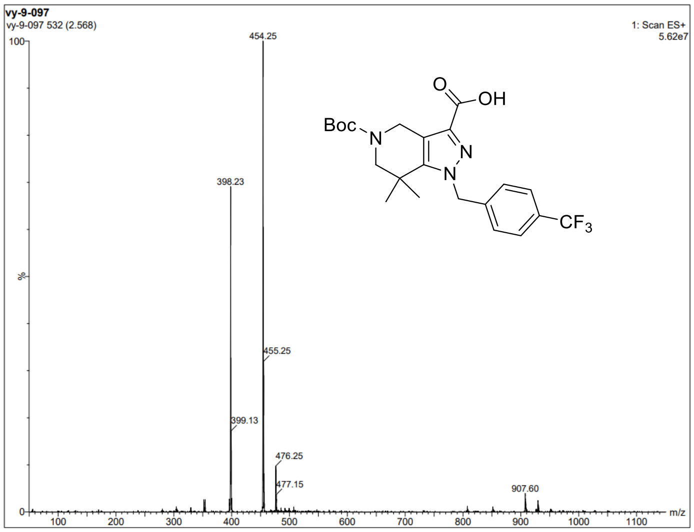

^{1}H NMR and LC-MS of compound 5

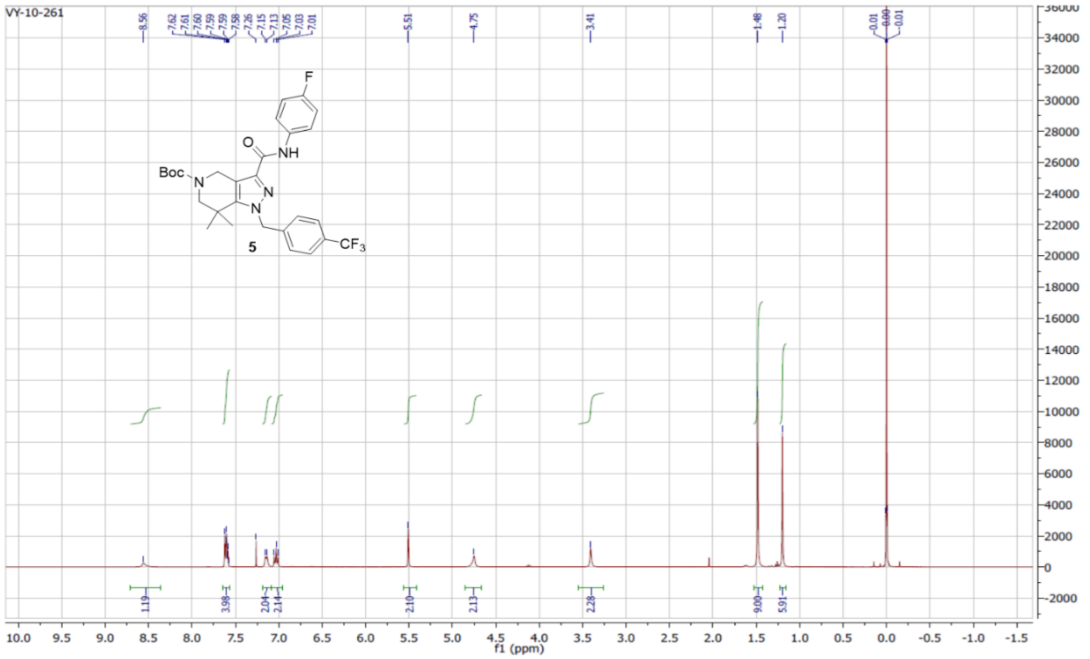

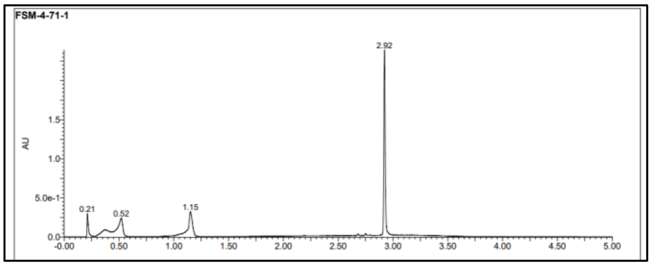

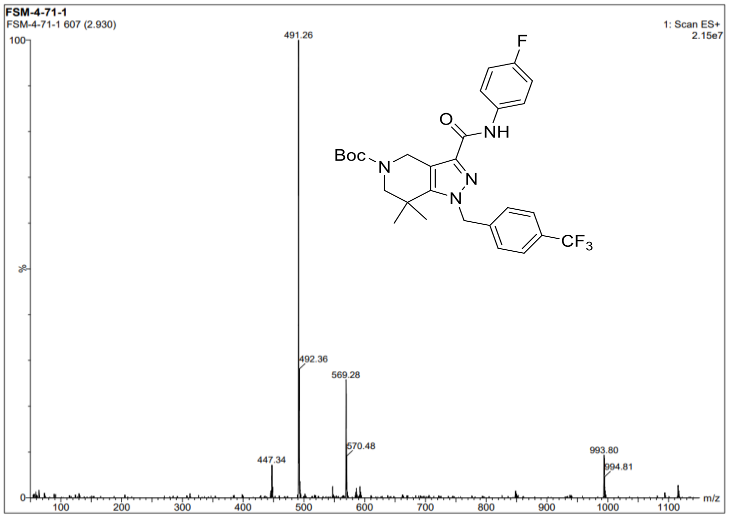

^{1}H NMR and LC-MS of compound 6

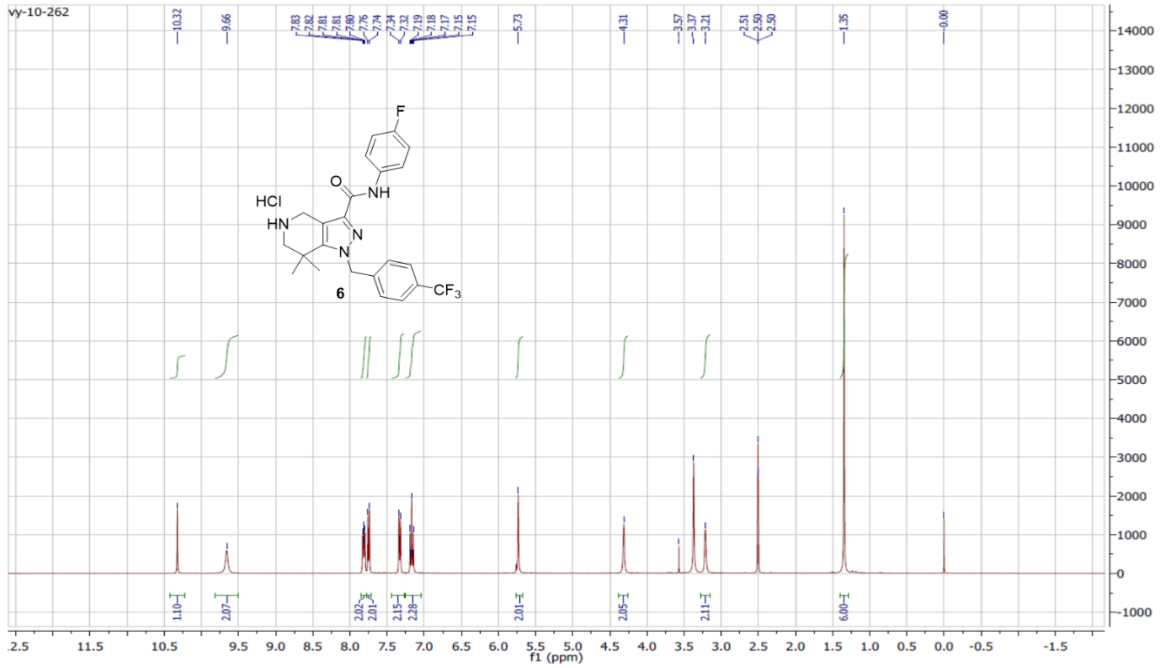

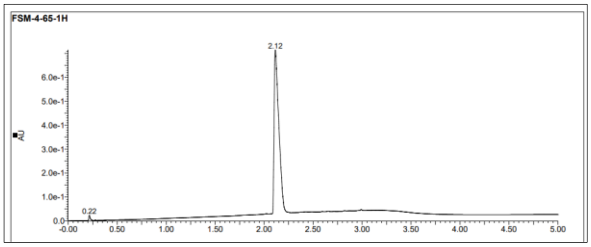

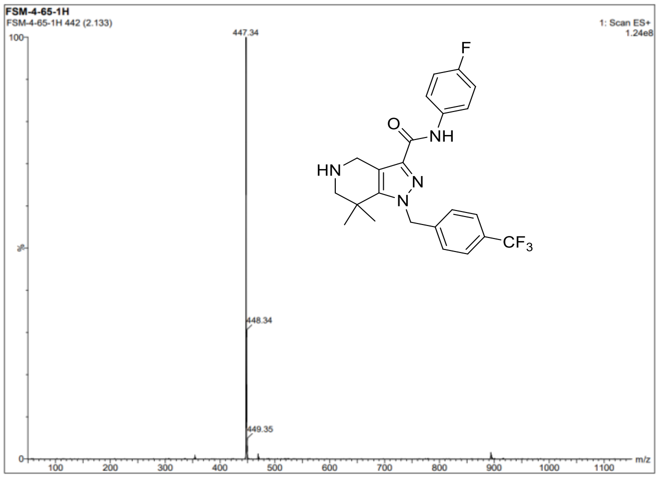

^{1}H NMR and LC-MS of compound FSM-3-002

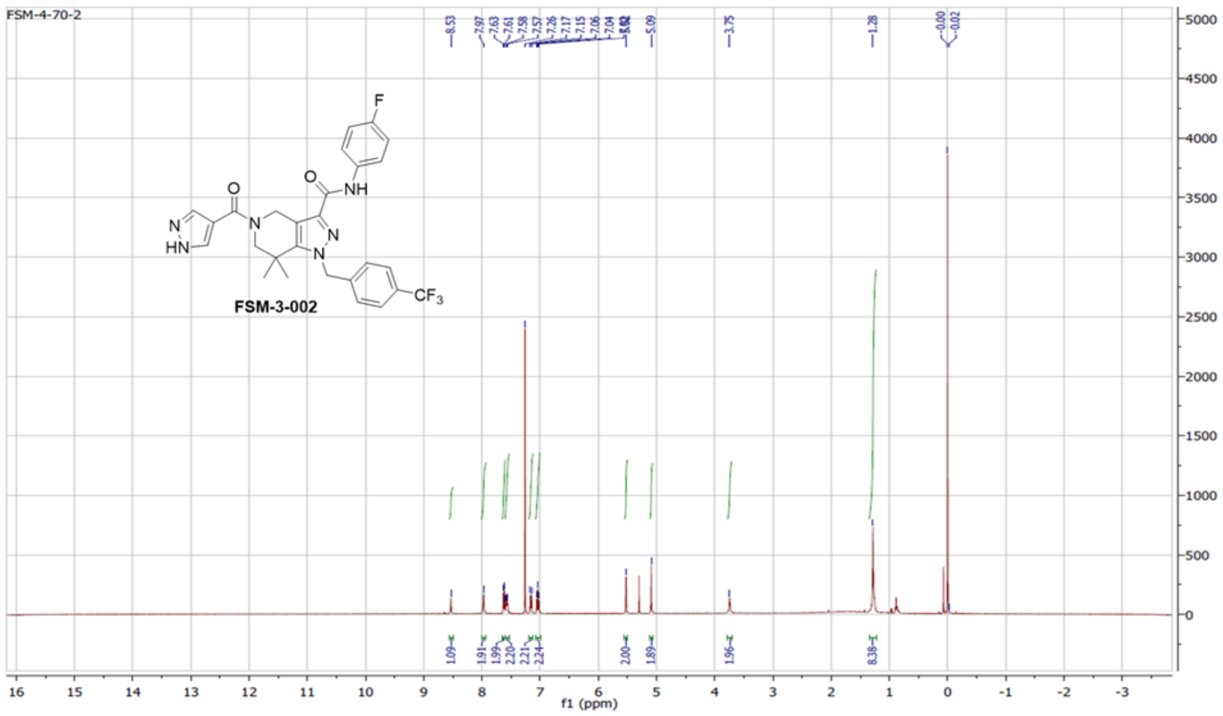

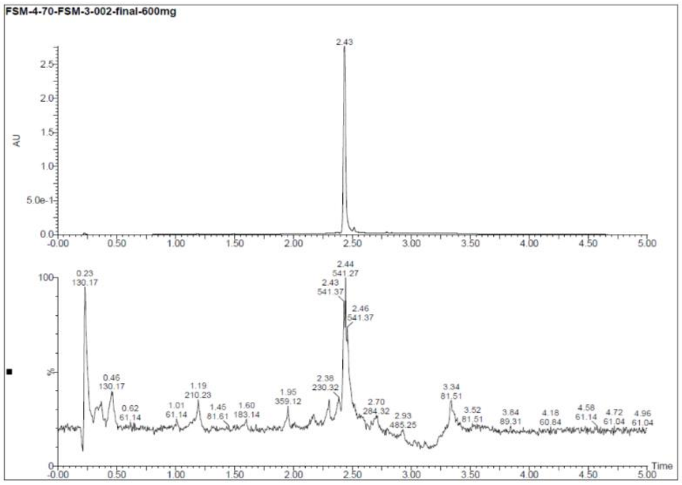

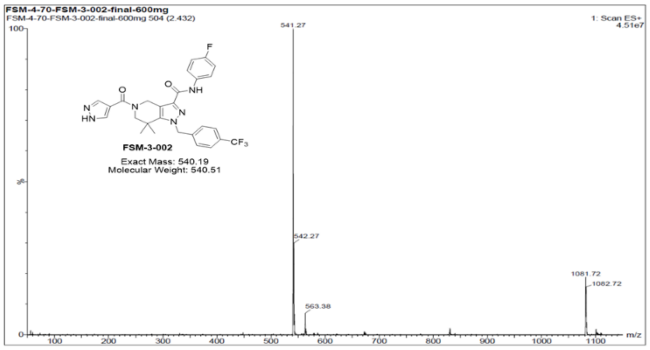

^{13}C NMR of compound FSM-3-002

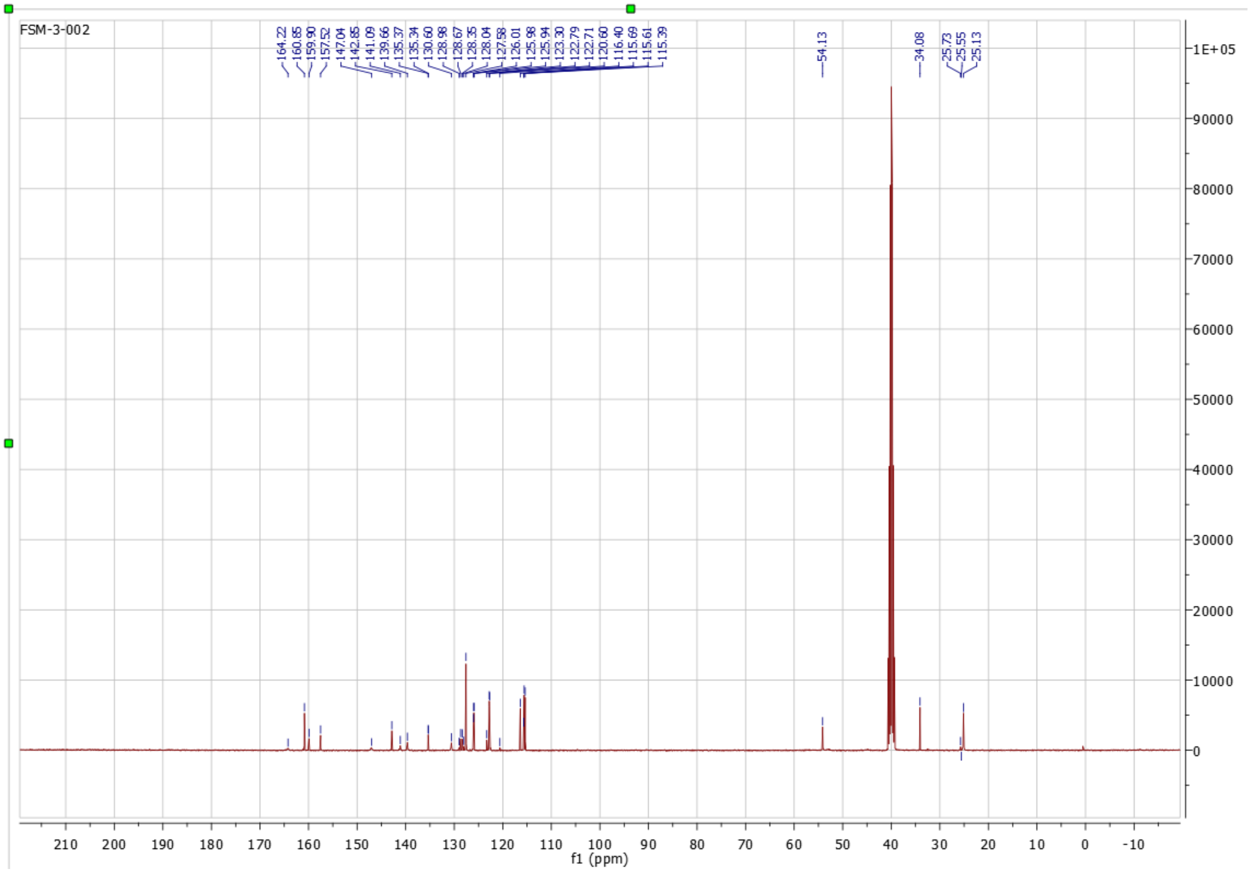

Intrinsic Clearance of FSM-3-002 and Midazolam Control in Mouse Liver Microsomes

	Microsomal Stability (Mouse, Compound Conc=0.5µM, n=1)		
**Control**	**Midazolam**		
**Test compound**	**Elimination rate constant (k) (min^{-1})**	**Half life (t1/2) (min)**	**Intrinsic Clearance (CLint) (mL/min/g liver)**
FSM-3-002	0.00472	147	0.453
Midazolam	0.395	1.76	37.9

Synthetic Scheme and Spectra Characterization for IDH1 Binding Probe IK-1-12

Scheme. Synthesis of IK-1-012

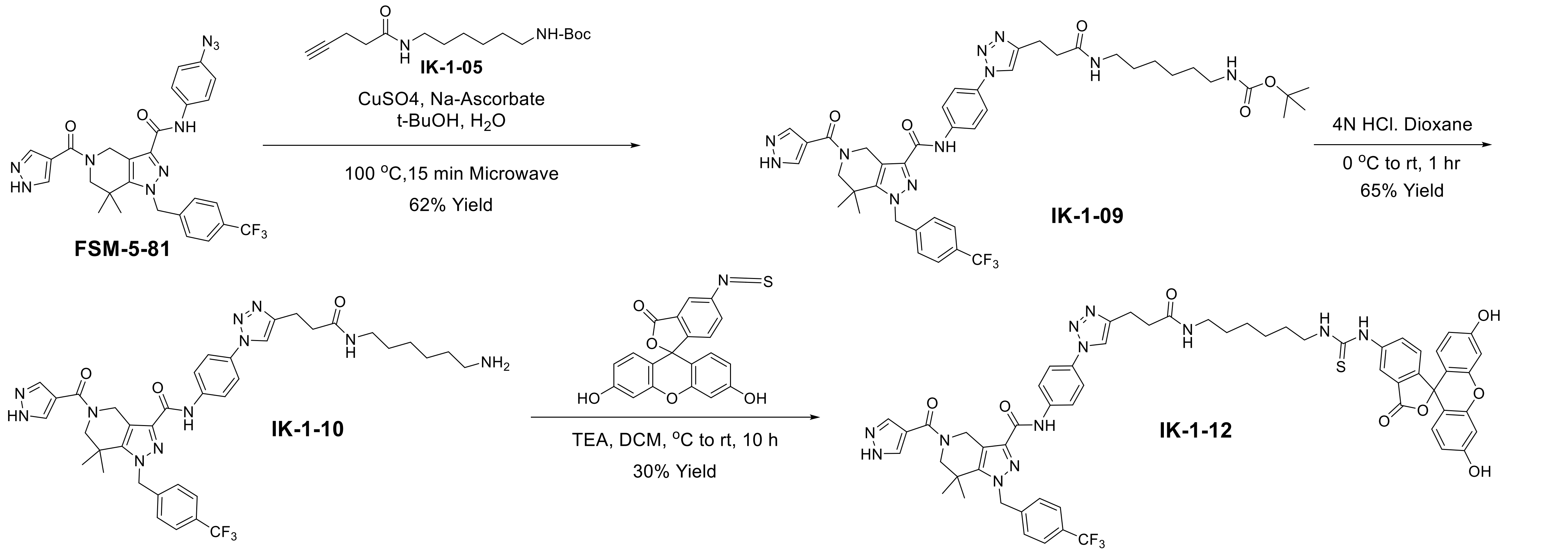

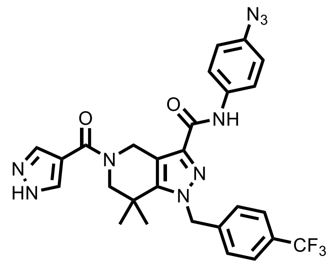

FSM-5-81
Molecular Weight: 563.55

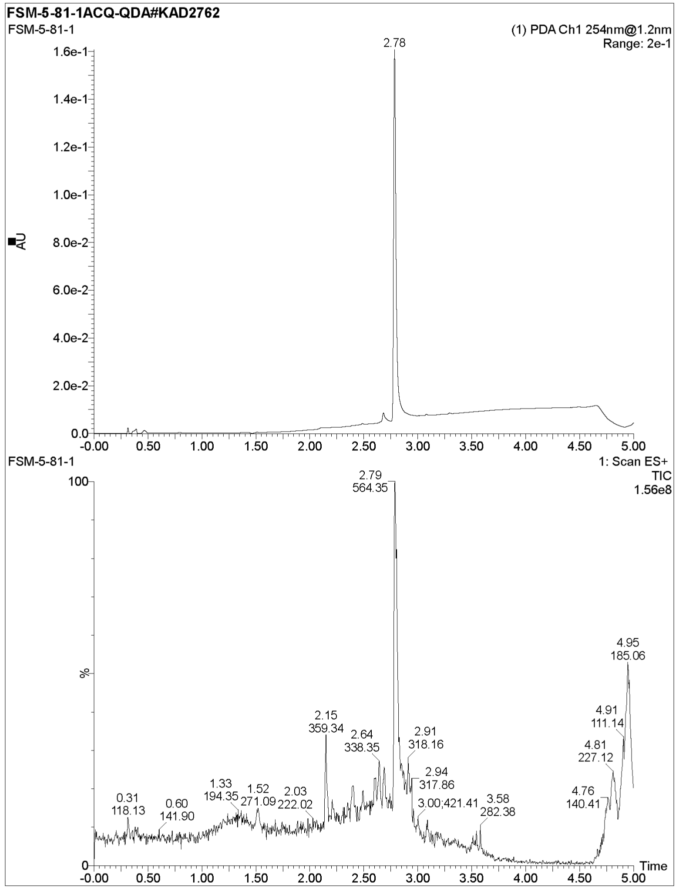

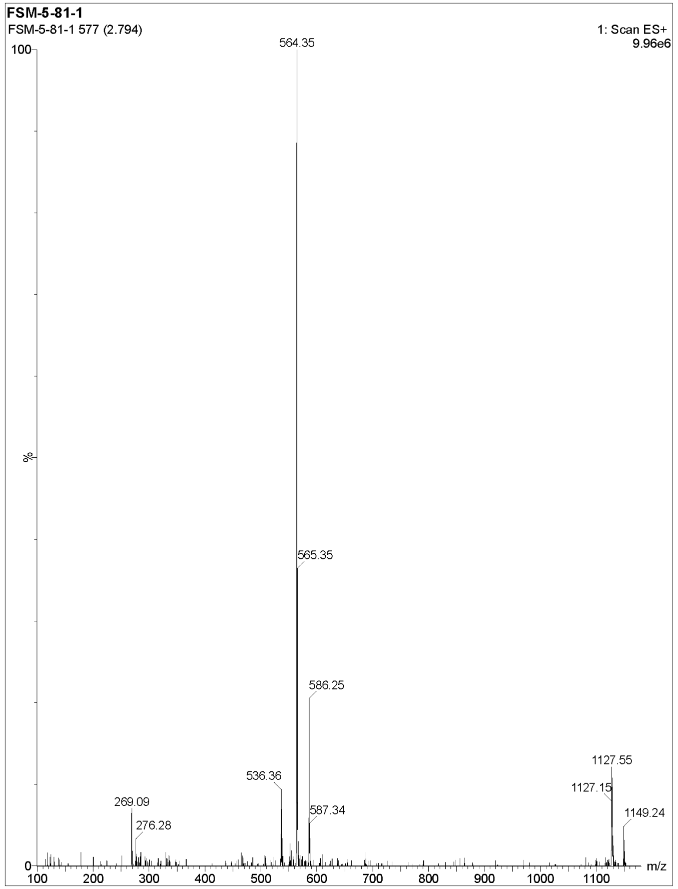

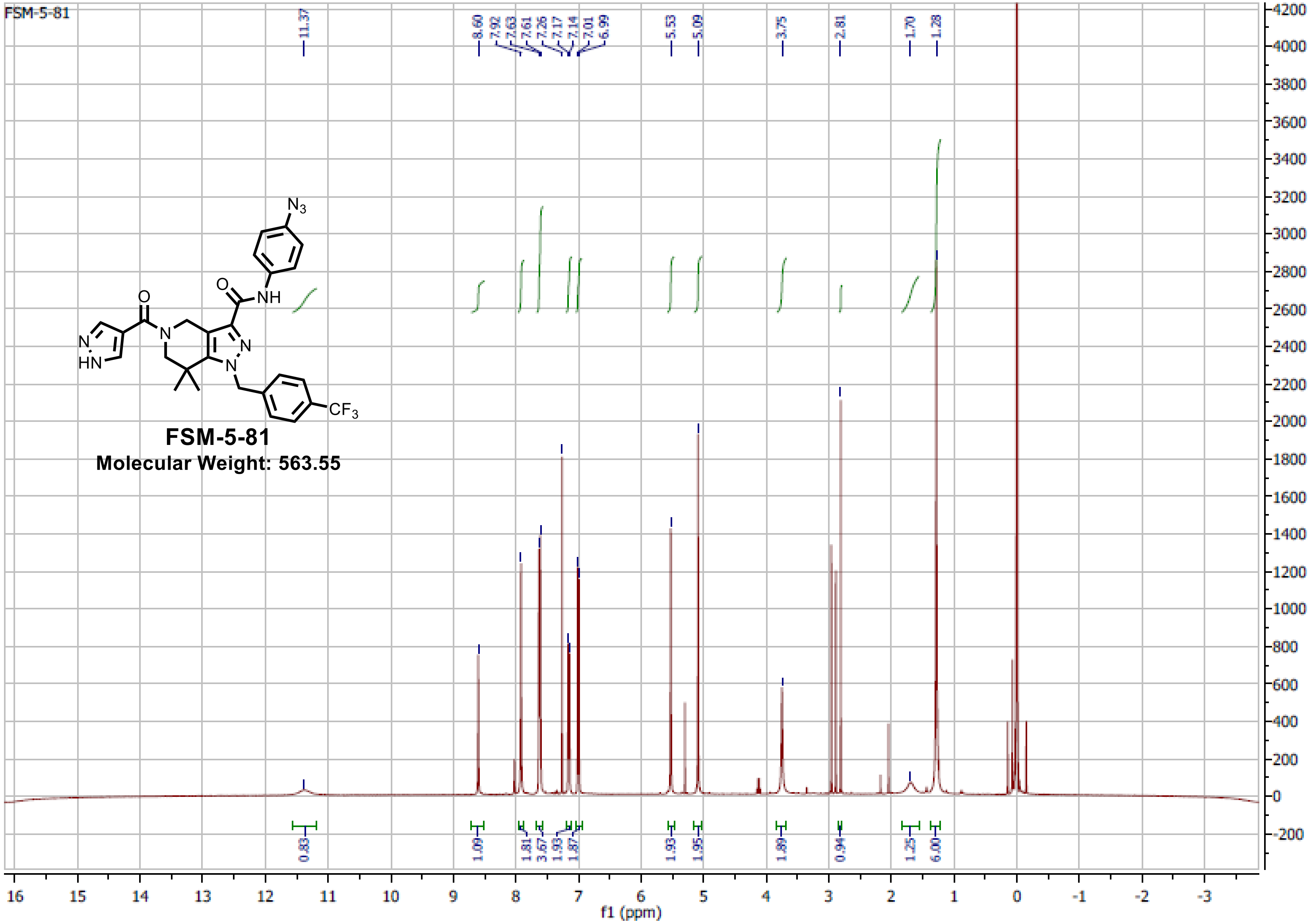
FSM-5-81
11.37
8.60
7.92
7.63
7.61
7.26
7.17
7.14
7.01
6.99
5.53
5.09
3.75
2.81
1.70
1.28
FSM-5-81
Molecular Weight: 563.55
0.83
1.09
1.81
3.67
1.93
1.87
1.93
1.95
1.89
0.94
1.25
6.00
f1 (ppm)

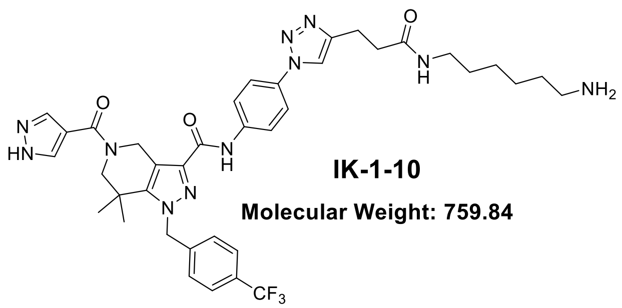

IK-1-10

Molecular Weight: 759.84

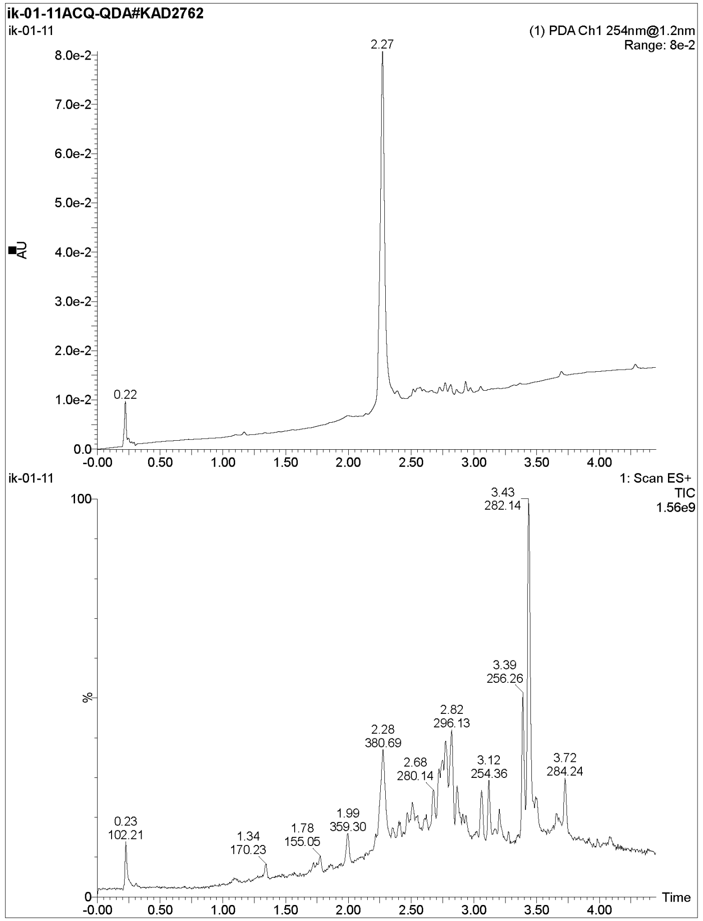

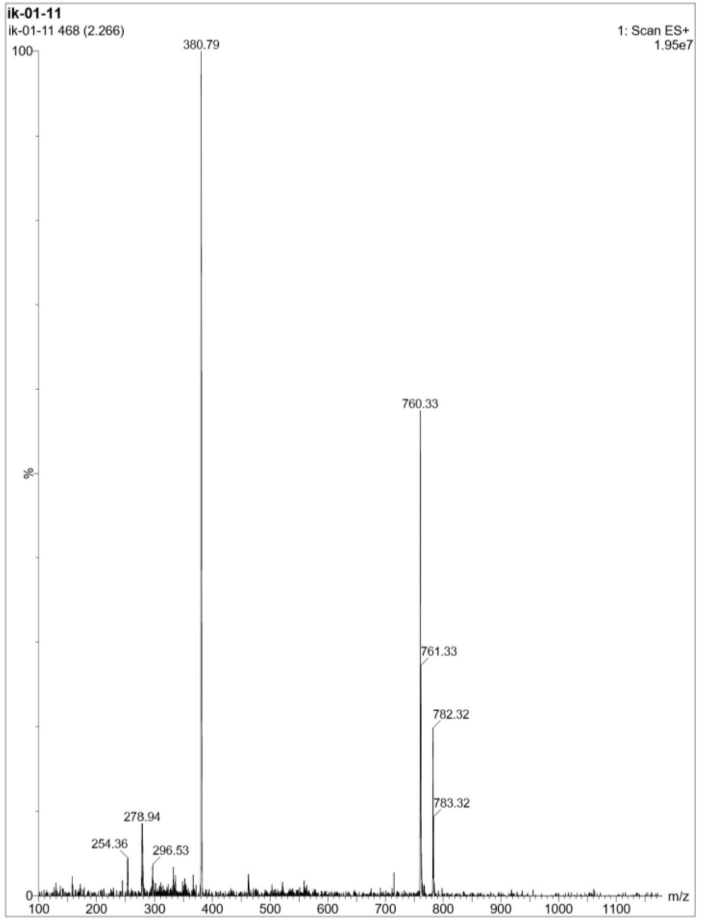

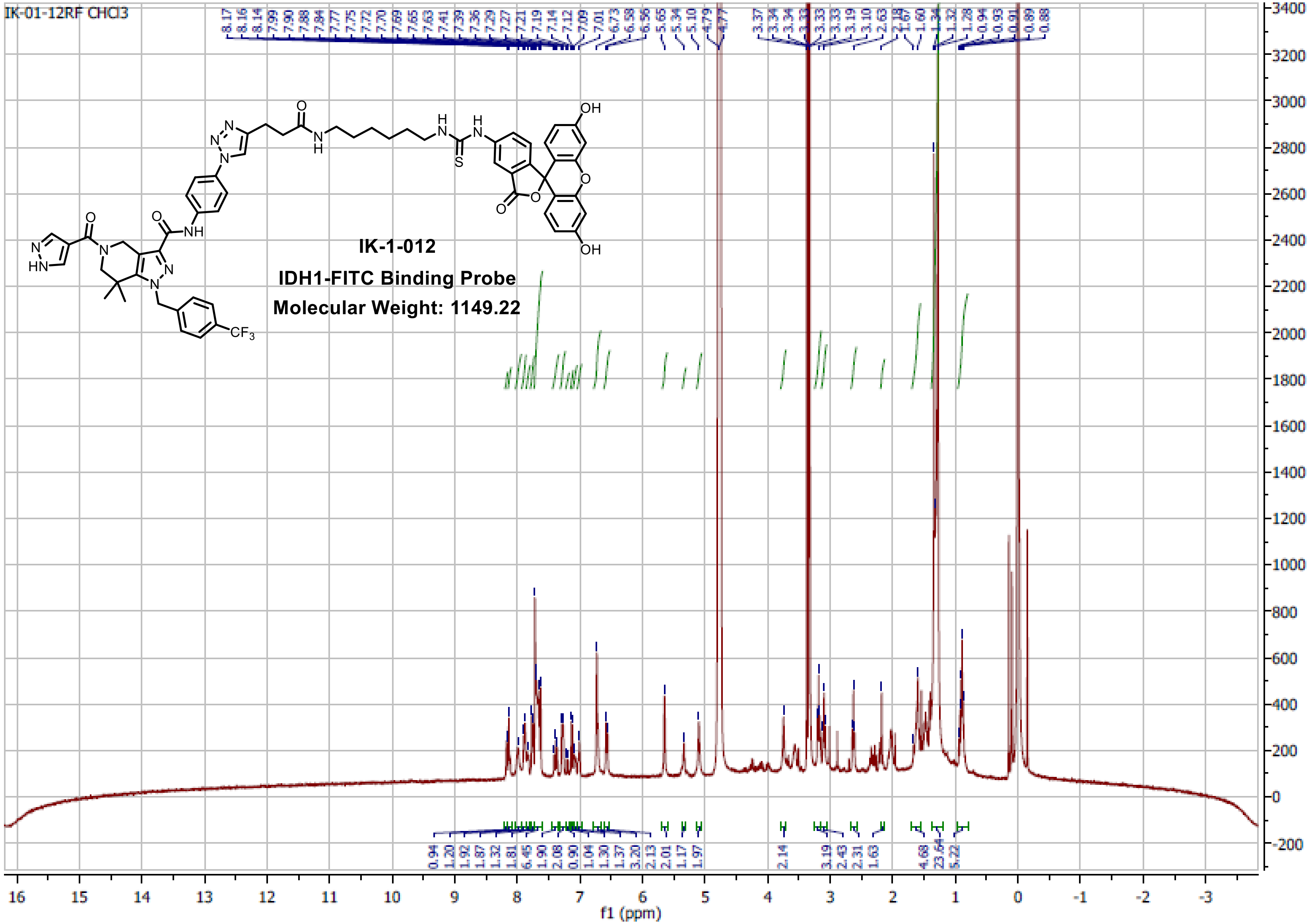
IK-01-12RF CHCl3
IK-1-012
IDH1-FITC Binding Probe
Molecular Weight: 1149.22
f1 (ppm)

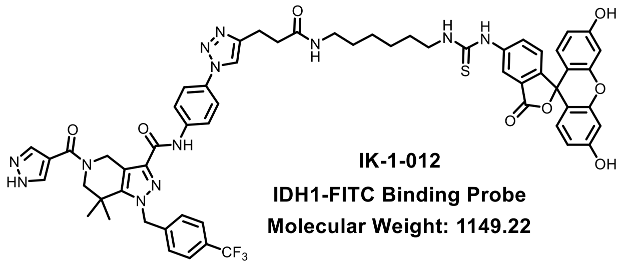

IK-1-012
IDH1-FITC Binding Probe
Molecular Weight: 1149.22

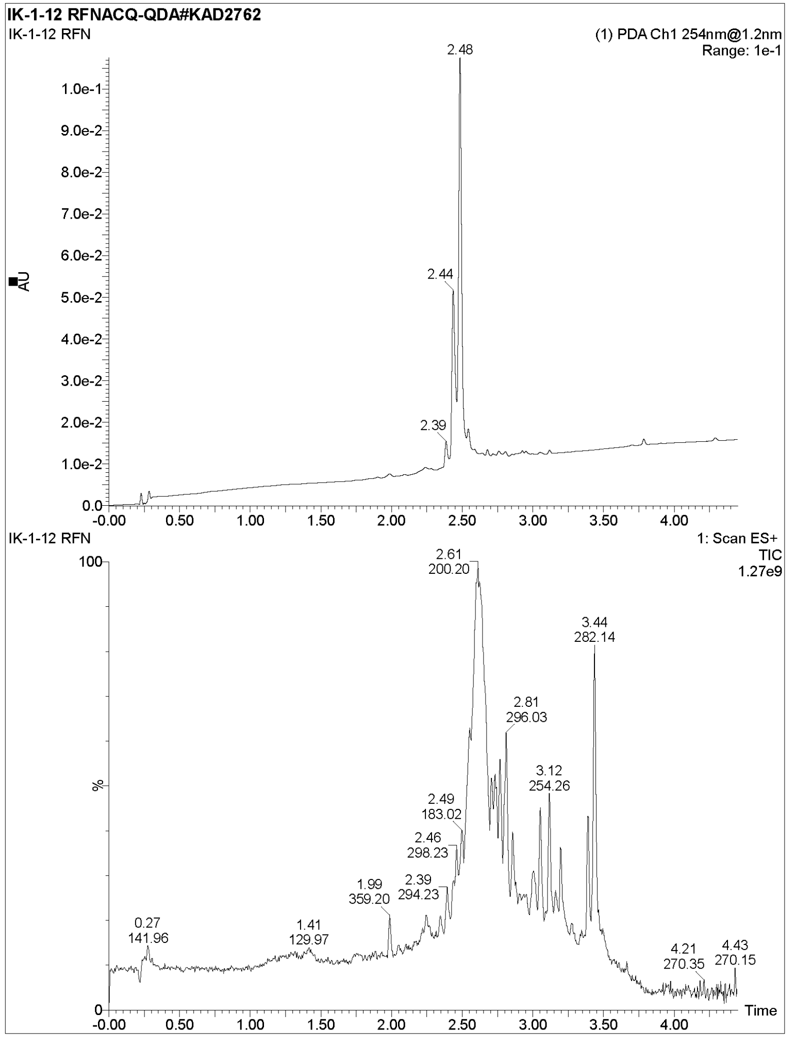

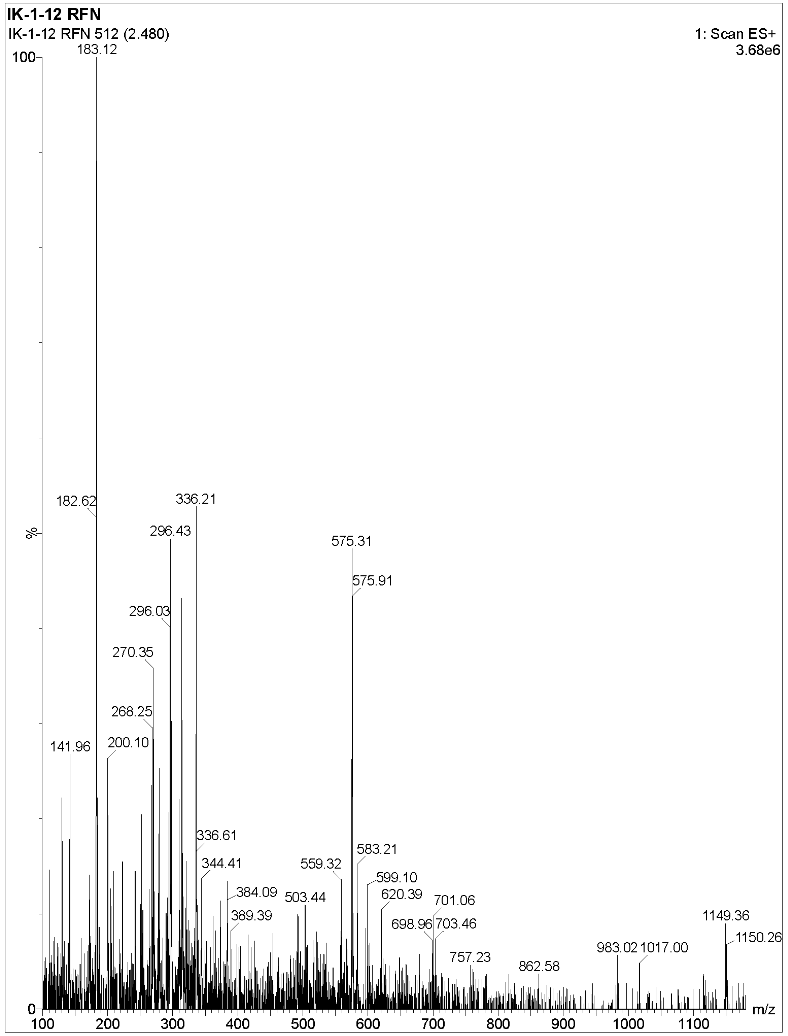

Supplement: Supplementary file 1 — FSM-3-002 synthesis, MLM stability and IK-1-12 scheme. [file 43018_2022_393_MOESM1_ESM.pdf]
