# Supplementary material for: Limited nutrient availability in the tumor microenvironment renders pancreatic tumors sensitive to allosteric IDH1 inhibitors
Source: Nat Cancer. 2022 Jun 9;3(7):852–65. doi: 10.1038/s43018-022-00393-y (PMC9325670; doi:10.1038/s43018-022-00393-y)
Supplement: Supplementary file 2 — Reporting Summary [file 43018_2022_393_MOESM2_ESM.pdf]

nature portfolio

Corresponding author(s): Jordan M. Winter

Last updated by author(s): Apr 26, 2022

Reporting Summary

Nature Portfolio wishes to improve the reproducibility of the work that we publish. This form provides structure for consistency and transparency in reporting. For further information on Nature Portfolio policies, see our Editorial Policies and the Editorial Policy Checklist.

Statistics

For all statistical analyses, confirm that the following items are present in the figure legend, table legend, main text, or Methods section.

n/a	Confirmed	
☐	☒	The exact sample size (*n*) for each experimental group/condition, given as a discrete number and unit of measurement
☐	☒	A statement on whether measurements were taken from distinct samples or whether the same sample was measured repeatedly
☐	☒	The statistical test(s) used AND whether they are one- or two-sided<br>*Only common tests should be described solely by name; describe more complex techniques in the Methods section.*
☒	☐	A description of all covariates tested
☒	☐	A description of any assumptions or corrections, such as tests of normality and adjustment for multiple comparisons
☐	☒	A full description of the statistical parameters including central tendency (e.g. means) or other basic estimates (e.g. regression coefficient) AND variation (e.g. standard deviation) or associated estimates of uncertainty (e.g. confidence intervals)
☐	☒	For null hypothesis testing, the test statistic (e.g. *F*, *t*, *r*) with confidence intervals, effect sizes, degrees of freedom and *P* value noted<br>*Give P values as exact values whenever suitable.*
☒	☐	For Bayesian analysis, information on the choice of priors and Markov chain Monte Carlo settings
☒	☐	For hierarchical and complex designs, identification of the appropriate level for tests and full reporting of outcomes
☒	☐	Estimates of effect sizes (e.g. Cohen's *d*, Pearson's *r*), indicating how they were calculated

Our web collection on statistics for biologists contains articles on many of the points above.

Software and code

Policy information about availability of computer code

Data collection	No software was used for data collection.
Data analysis	Supporting data are available within the article or its Supplementary files. qPCR acquisition were captured using Bio-Rad CFX96 and analyzed using Bio-Rad CFX Manager 2.0 software. Western blot densities were quantified using Image Studio Software Version 5.2.5. Blots were probed with secondary antibodies customized for the Odyssey Imaging system (Thermo Fisher Scientific, 32106). GC-MS data were analyzed using the MSD ChemStation Software, version: F.01.03.2357 (Agilent Technologies). Agilent MassHunter Workstation Software LC/MS Data Acquisition for 6400 Series Triple Quadrupole MS with Version B.08.02 was used for compound optimization and sample data acquisition. Agilent MassHunter Quantitative Analysis B.09.00 QqQ software was used to integrate and quantitate areas. Statistical analysis using GraphPad Prism 9.

For manuscripts utilizing custom algorithms or software that are central to the research but not yet described in published literature, software must be made available to editors and reviewers. We strongly encourage code deposition in a community repository (e.g. GitHub). See the Nature Portfolio guidelines for submitting code & software for further information.

Data

Policy information about availability of data

All manuscripts must include a data availability statement. This statement should provide the following information, where applicable:

- Accession codes, unique identifiers, or web links for publicly available datasets
- A description of any restrictions on data availability
- For clinical datasets or third party data, please ensure that the statement adheres to our policy

Open source software were used for RNA-seq analysis: FastQC (https://www.bioinformatics.babraham.ac.uk/projects/fastqc/), Trim Galore (http://

www.bioinformatics.babraham.ac.uk/projects/trim_galore/), R (v 3.6.3 and v 3.4.2), STAR (v 2.7.0e), DESeq2 (v 1.26.0), and RSEM (v 1.3.2). RNA sequencing data was deposited into Gene Expression Omnibus with accession number GSE161073. DNA sequences were deposited into GenBank with accession numbers ON022872-ON022876. Metabolomics has been deposited to the EMBL-EBI MetaboLights database with the accession number MTBLS4722. Source data for Fig. 1-8 and Extended Data Fig. 1-4, 6-8 have been provided as Source Data files. All other data supporting the findings of this study are available from the corresponding author on reasonable request.

Field-specific reporting

Please select the one below that is the best fit for your research. If you are not sure, read the appropriate sections before making your selection.

☒ Life sciences ☐ Behavioural & social sciences ☐ Ecological, evolutionary & environmental sciences

For a reference copy of the document with all sections, see nature.com/documents/nr-reporting-summary-flat.pdf

Life sciences study design

All studies must disclose on these points even when the disclosure is negative.

Sample size	Sample sizes were not determined based on statistical power determination, but were based on our experience with these assays and reported in previous studies (Cancer Res. 77(16), 2017; Science 366(6468), 2019).
Data exclusions	No data were excluded from the analyses, except one sample from metabolomics since its total metabolite levels was below threshold.
Replication	Experiments were repeated independently at least three times, as indicated in the figure legends. It also has mentioned in the source data files. While data shown in a minority of panels are from a representative experiment, the number of independent experiments that reproduced the finding is also indicated in the figure legends.
Randomization	Mice with confirmed tumors were then randomized to the indicated treatment arms. No formal randomization techniques were used; however, animals were allocated randomly to the experiments. The investigators were not blinded to allocation during experiments because treatments used in the study made it difficult to blind. For in vitro studies, the experiments were assigned randomly to control and experimental groups in different plates (i.e., 6-well or 96-well plates purchased for various vendors) and formats (i.e., at different positions in the plate) performed by different investigators for a number of independent replicates to prevent experimental bias. We often purchase the reagents from different vendors to evaluate the reproducibility of our outcomes.
Blinding	The investigators were not blinded to allocation during experiments because treatments used in the study made it difficult to blind.

Reporting for specific materials, systems and methods

We require information from authors about some types of materials, experimental systems and methods used in many studies. Here, indicate whether each material, system or method listed is relevant to your study. If you are not sure if a list item applies to your research, read the appropriate section before selecting a response.

Materials & experimental systems

n/a	Involved in the study	
☐	☒	Antibodies
☐	☒	Eukaryotic cell lines
☒	☐	Palaeontology and archaeology
☐	☒	Animals and other organisms
☒	☐	Human research participants
☒	☐	Clinical data
☒	☐	Dual use research of concern

Methods

n/a	Involved in the study	
☒	☐	ChIP-seq
☐	☒	Flow cytometry
☒	☐	MRI-based neuroimaging

Antibodies

Antibodies used	Ki-67, Cat. #9027, Rabbit Monoclonal Antibody, Clone D2H10, Cell Signaling, 1:400, IHC.<br>Cleaved Caspase-3, Cat. #9579, Rabbit Monoclonal Antibody (Asp 175)(D3E9), Cell Signaling, 1:250, IHC.<br>IDH1, GT1521, Mouse Monoclonal Antibody (GT1521), Catalog (# MA5-27759, Invitrogen; # ab184615, abcam), 1:1000, WB.<br>alpha Tubuin, 11224-1-AP, Rabbit Polyclonal Antibody, 1:5000, WB.<br>beta Actin, sc-47778, Santa Cruz Tech, 1:5000, WB.
Validation	All antibodies are commercially available, and have been validated by previously published studies. For instance:<br>- alpha Tubulin: Dev. Cell 48(3), 2019; Autophagy 15(3), 2019.<br>- IDH1: Cancer Res, 77(16), 2017.<br>- beta Actin: Mol Cancer Res, 15(4), 2017.

Eukaryotic cell lines

Policy information about cell lines

Cell line source(s)	MiaPaCa-2, PANC-1, Hs766T, HCT116, H460, HEK293 cells were purchased from ATCC. KPC K8484 cells (KrasG12D/+; Trp53R172H/+; Pdx1-Cre) were obtained from the laboratory of Darren Carpizo (Yu et al., Clin Cancer Res, 2018).
Authentication	All cell lines were authenticated by ATCC via STR profiling.
Mycoplasma contamination	All cell lines were confirmed to be mycoplasma-free by MycoAlert detection kit (Lonza).
Commonly misidentified lines (See ICLAC register)	No commonly misidentified cell lines were used.

Animals and other organisms

Policy information about studies involving animals; ARRIVE guidelines recommended for reporting animal research

Laboratory animals	Six-to-eight week-old, female, athymic nude mice (Foxn1 nu/nu) were purchased from Harlan Laboratories.<br>Luciferase-expressing KPC K8484 cells were injected into the pancreas of C57BL/6 mice at 12 weeks of age. Equal number of male and female mice were used for survival analysis.<br>KPC (KrasG12D/+; Trp53R172H/+; Pdx1-Cre) mice were bred in-house at the CRUK Beatson Institute, maintained in conventional caging and environmental enrichment. Both genders were used in the study.<br>Six-to-eight-week-old, male and female, Tamoxifen-inducible KPlox/loxC (KrasG12D/+; Trp53lox/lox; Pdx1-Cre/Esr1) mice were purchased from The Jackson Laboratory (#032429). Both genders were used in the study.
Wild animals	The study did not involve wild animals.
Field-collected samples	No field-collected samples were used in this study.
Ethics oversight	All animal experiments were conducted in accordance with a protocol approved by Institutional Animal Care and Use Committee at Case Western Reserve University. All experiments with KPC mice were performed under a UK Home Office license and approved by the University of Glasgow Animal Welfare and Ethical Review Board.

Note that full information on the approval of the study protocol must also be provided in the manuscript.

Flow Cytometry

Plots

Confirm that:

- [ ] The axis labels state the marker and fluorochrome used (e.g. CD4-FITC).
- [ ] The axis scales are clearly visible. Include numbers along axes only for bottom left plot of group (a 'group' is an analysis of identical markers).
- [ ] All plots are contour plots with outliers or pseudocolor plots.
- [ ] A numerical value for number of cells or percentage (with statistics) is provided.

Methodology

Sample preparation	Cells were trypsinized, and EGFP-expressing cells were sorted via fluorescence-activated cell sorting (FACS) analysis
Instrument	FACS Aria II, BD Biosciences
Software	*Describe the software used to collect and analyze the flow cytometry data. For custom code that has been deposited into a community repository, provide accession details.*
Cell population abundance	*Describe the abundance of the relevant cell populations within post-sort fractions, providing details on the purity of the samples and how it was determined.*
Gating strategy	*Describe the gating strategy used for all relevant experiments, specifying the preliminary FSC/SSC gates of the starting cell population, indicating where boundaries between "positive" and "negative" staining cell populations are defined.*

- [ ] Tick this box to confirm that a figure exemplifying the gating strategy is provided in the Supplementary Information.
