# Supplementary figures and images for: Limited nutrient availability in the tumor microenvironment renders pancreatic tumors sensitive to allosteric IDH1 inhibitors
Source: Nat Cancer. 2022 Jun 9;3(7):852–65. doi: 10.1038/s43018-022-00393-y (PMC9325670; doi:10.1038/s43018-022-00393-y)

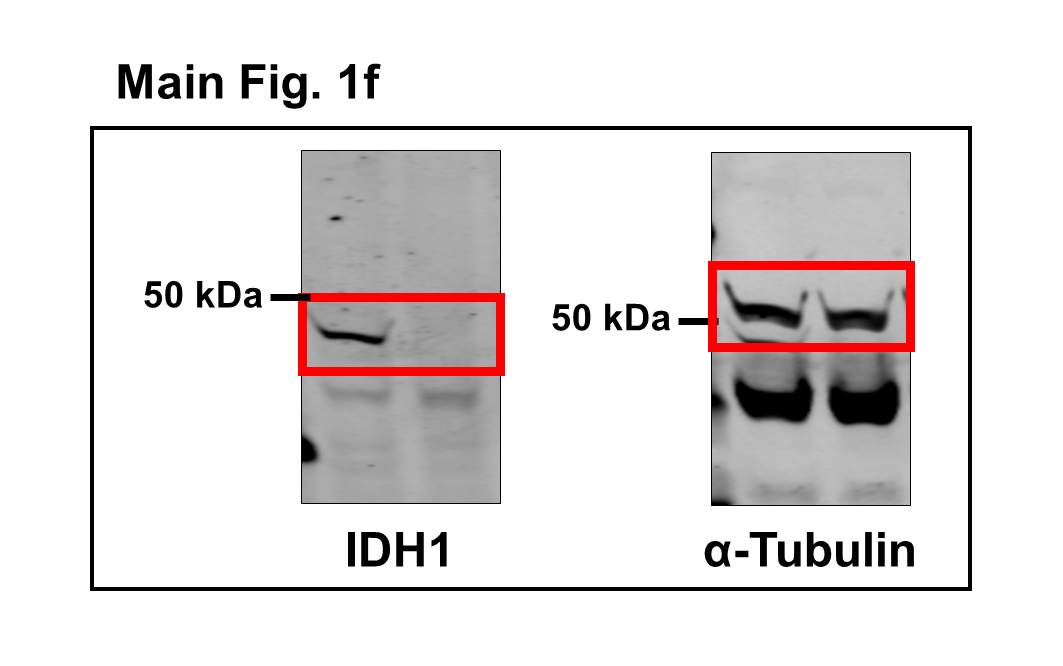

Supplement: Source Data Fig. 1 — Uncropped blots. [file 43018_2022_393_MOESM4_ESM.tif]

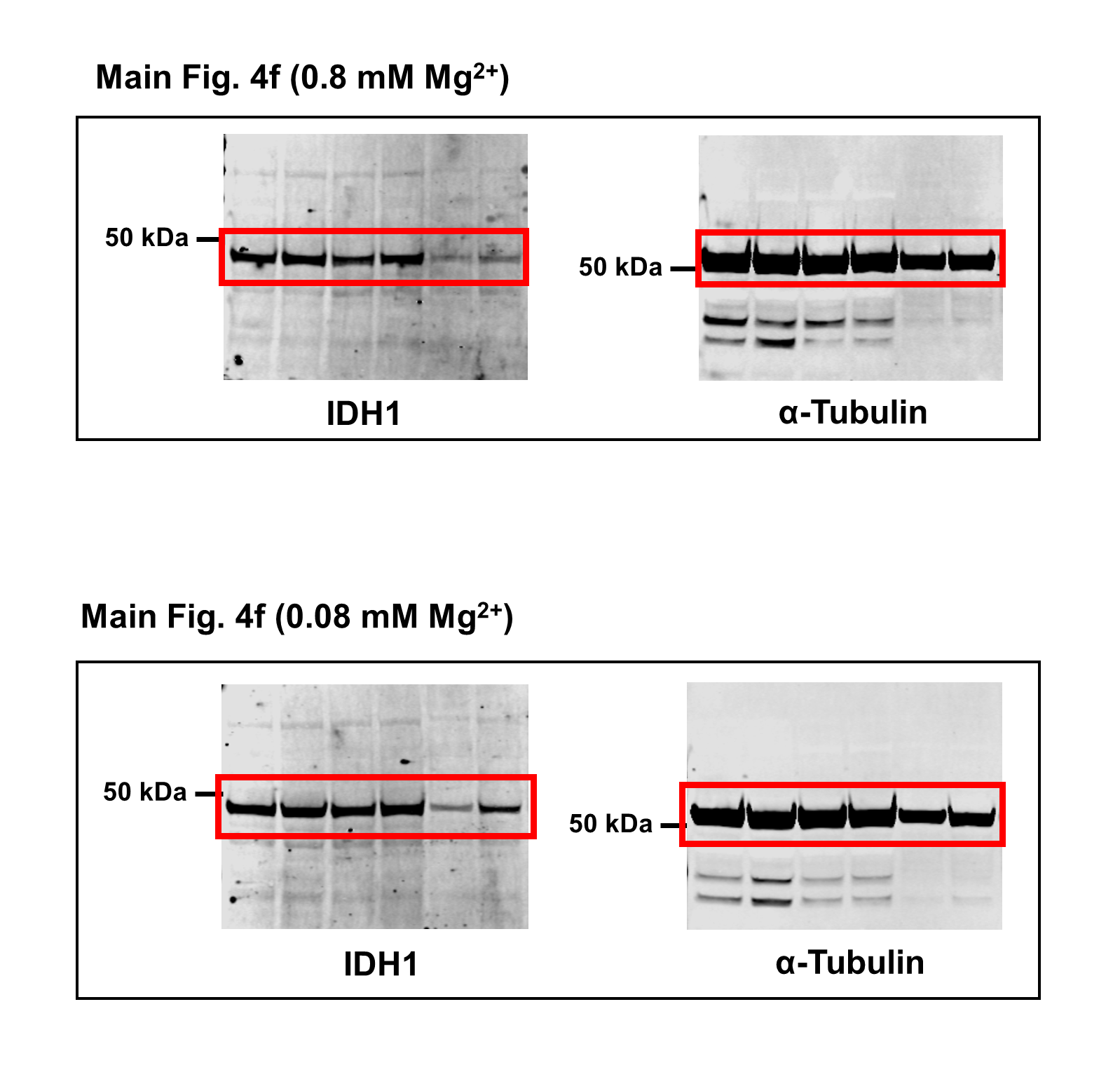

Supplement: Source Data Fig. 4 — Uncropped blots. [file 43018_2022_393_MOESM8_ESM.tif]

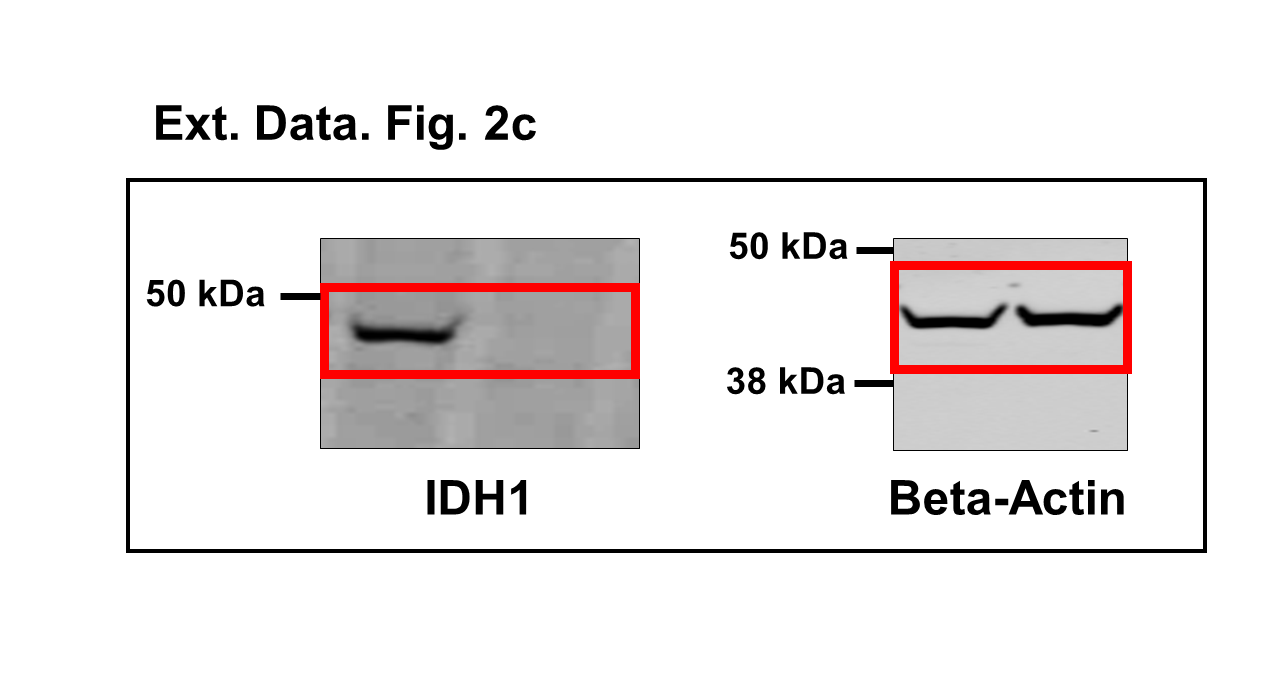

Supplement: Source Data Extended Data Fig. 2 — Uncropped blots. [file 43018_2022_393_MOESM15_ESM.tif]

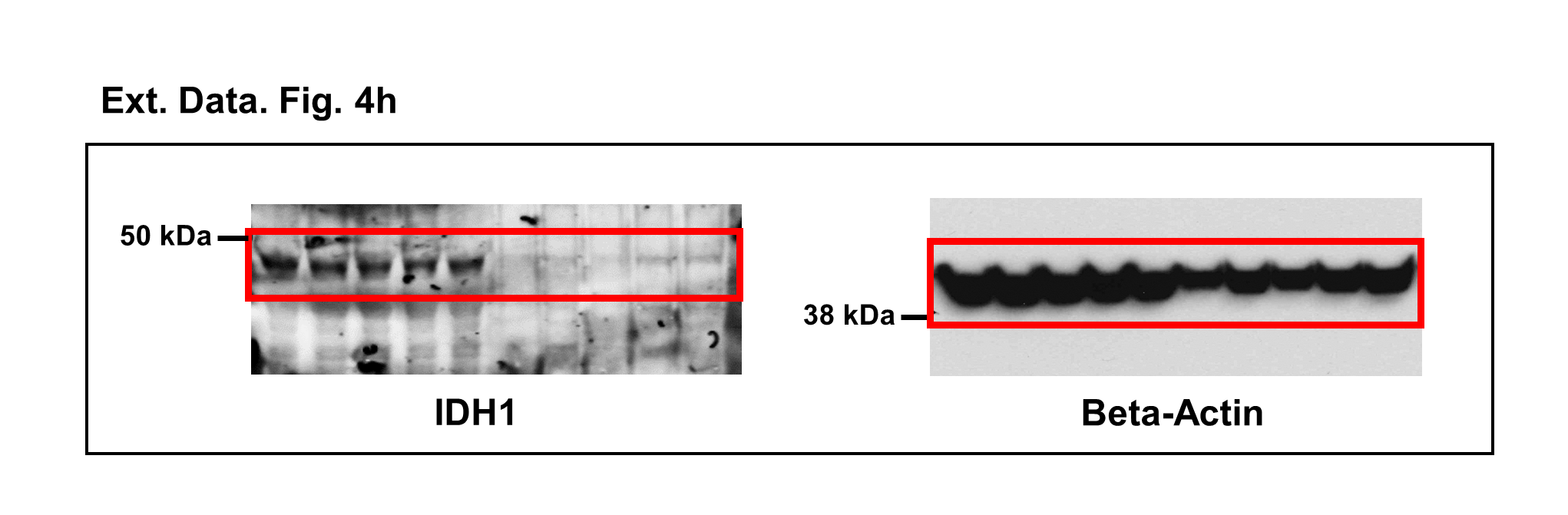

Supplement: Source Data Extended Data Fig. 4 — Uncropped blots. [file 43018_2022_393_MOESM18_ESM.tif]
